# Characteristics of influenza, SARS-CoV-2, and RSV surveillance systems that utilise ICD-coded data: a systematic review

**DOI:** 10.7189/jogh.15.04177

**Published:** 2025-05-23

**Authors:** Eldad Agyei-Manu, Nadege Atkins, Madhurima Nundy, Christa St-Jean, Alice Gornall-Wick, Emma Birley, Udani De Silva, Prerna Krishan, Laura Vokey, Marshall F Dozier, Emilie McSwiggan, Ruth McQuillan, Evropi Theodoratou, Ting Shi, UNCOVER Applied Evidence Synthesis group

**Affiliations:** 1 Centre for Population Health Sciences, Usher Institute, University of Edinburgh, Edinburgh, Scotland, UK; 2 EAM Consult, Sunyani, Ghana; 3 Information Services, University of Edinburgh, Edinburgh, Scotland, UK; 4 Centre for Global Health, Usher Institute, University of Edinburgh, Edinburgh, Scotland, UK; 5 Cancer Research UK Edinburgh Centre, MRC Institute of Genetics and Cancer, University of Edinburgh, Edinburgh, Scotland, UK; 6 Centre for Medical Informatics, Usher Institute, University of Edinburgh, Edinburgh, Scotland, UK

## Abstract

**Background:**

Some surveillance systems for influenza, severe acute respiratory syndrome coronavirus 2 (SARS-CoV-2), and respiratory syncytial virus (RSV) utilise International Classification of Diseases (ICD)-coded data and are useful for analysing trends and enhancing quick, evidence-based decisions against the epidemic potential that threatens global health security. With variations in the design of systems globally, the World Health Organization requested a systematic review to identify key characteristics of influenza, SARS-CoV-2, and RSV surveillance systems that utilise ICD-coded data, and to assess their performance.

**Methods:**

We searched EMBASE, MEDLINE, and Global Health to identify relevant studies reporting on influenza, SARS-CoV-2, and RSV surveillance systems that use ICD-coded data. We independently assessed studies for the ICD codes used, their statistical estimates and limitations. We appraised included studies using Joana Briggs Institute’s critical appraisal tools and synthesised using narrative synthesis.

**Results:**

We identified 77 studies, reporting on 71 surveillance systems – 33 systems recorded surveillance data only, 15 systems recorded burden of disease data only, and 23 systems recorded both surveillance and burden of disease data. Surveillance systems utilised ICD-10 codes (75%), ICD-9 codes (22%), or both (3%). ICD-10 codes J09 and J10 were frequently used for influenza, U07.1 for COVID-19, and B97.4, J12.1, J20.5, and J21.0 for RSV. ICD-9 codes 487 and 488 were mostly used for influenza, and codes 466.11 and 480.1 for RSV. ICD-10 codes had low-to-moderate sensitivity (6.60–79.87%) and high specificity (97.40–99.72%) for influenza, low-to-high sensitivity (30.00–98.4%) and specificity (39.50–99.80%) for COVID-19, and low-to-high sensitivity (6.00–99.80%) and specificity (12.10–100.00%) for RSV. ICD-9 codes had low sensitivity (45.60%) and high specificity (97.90%) for influenza. Underestimation of infections or mortality attributable to influenza, SARS-CoV-2, or RSV is a major limitation to using ICD-coded data across surveillance systems.

**Conclusions:**

The performance of ICD codes for syndromic- or disease-specific surveillance remains inconclusive, although using only ICD-coded data within these systems may underestimate influenza, SARS-CoV-2, or RSV-attributable morbidity and mortality. Future studies should assess the accuracy of ICD code combinations for surveillance of influenza, SARS-CoV-2, and RSV.

International Classification of Diseases (ICD), a coding system maintained and published by the World Health Organization (WHO), is regarded as the global standard for diagnostic health information. It provides vital information on the prevalence, causes and effects of human illnesses and mortality worldwide using data reported by health officials and offers consistent language for reporting, monitoring, and documenting diseases [1]. This further allows for data to be shared and compared globally in a uniform and standard manner across hospitals, nations, and regions over periods of time. The use of the ICD also facilitates more efficient data collection, storage, analysis and use in evidence-based decision-making [2].

WHO has developed and coordinated the global epidemiological and virological surveillance of influenza through its Global Influenza Surveillance and Response System, since 1952, using standardised case definitions including influenza-like illness (ILI) and severe acute respiratory infection (SARI) surveillance for syndromic illness. An ILI case is defined as ‘an acute respiratory infection with a fever of ≥38°C and a cough, and with an onset within the last 10 days,’ whereas a SARI case is defined as ‘an acute respiratory infection with history of fever or a measured fever of ≥38°C and a cough, with an onset within the last 10 days, and requires hospitalisation’ [3]. The 2013 WHO Global Epidemiological Surveillance Standards for Influenza [4] recommends using a sentinel surveillance system for ILI and SARI surveillance. This system is regarded as an efficient method for the systematic collection of high-quality, patient-related epidemiological and virological data on a routine basis from a limited number of surveillance sites. It assists public health decision-makers, health care providers and policymakers in health care decision-making and programme management.

While the WHO has not formally undertaken a critical assessment of the relationship between the clinical syndromes of ILI and SARI and the ICD codes used, it has acknowledged that the use of automated ICD-coded data, along with laboratory confirmation of influenza, could be useful in supplementing data obtained from SARI and ILI sentinel surveillance systems, although this approach is liable to inherent limitations and validation requirements [4]. Many health systems regularly store summary data on respiratory diseases from electronic data reporting systems that have information coded according to the ICD system [5]. ICD-code-based syndromic surveillance for ILI and SARI could be useful in informing and designing prevention and treatment policies, such as immunisation planning programmes.

While research has begun to show that surveillance systems that utilise ICD-coded data can enable timely, reliable information to be produced regarding respiratory viruses, and that this can support quick, evidence-based decision-making [6,7], there is a gap in the critical synthesis of the characteristics and performance of these systems. In this review, we aimed to identify key characteristics of influenza, respiratory syncytial virus (RSV), and severe acute respiratory syndrome coronavirus 2 (SARS-CoV-2) surveillance systems that utilise ICD-coded data, and assess their performance.

## METHODS

Prior to conducting this review, we developed a study protocol based on the PRISMA-P 2015 guidelines (Appendix S1 in the **Online Supplementary Document**) and later followed the PRISMA 2020 guidelines in reporting the study [8]. We systematically searched three databases (EMBASE, Ovid MEDLINE, and Global Health) to identify relevant studies. The development and adaptation of the search strategy for each database was guided by the University of Edinburgh Information Specialist (MFD) (Appendix S2 in the **Online Supplementary Document**). We ran an initial search on 11 January 2023 and a second search to update the evidence on 3 June 2024. We imported studies to Covidence (Covidence, Melbourne, Victoria, Australia) [9], and duplicates were automatically removed. Two independent reviewers from a group of nine reviewers (EAM, NA, MN, CS, AGW, EB, UDS, PK, and LV) performed the title and abstract screening and full-text screening to identify eligible papers. Any disagreement on study eligibility at both the title and abstract and full-text screening stages was resolved through discussion or consultation with a third reviewer (RM, ET, and TS). We included studies reporting on surveillance systems using ICD-coded data (surveillance data, burden of disease data, vaccine effectiveness data) for human influenza, RSV and SARS-CoV-2. Studies that reported on non-ICD-coded data and those in languages other than English were excluded from this review (**Table 1**).

**Table 1:** Eligibility criteria

Inclusion criteria	Exclusion criteria
Studies reporting on surveillance systems utilising ICD-coded data	Studies reporting on surveillance systems utilising non-ICD-coded data
Studies reporting on human influenza, SARS-CoV-2, and RSV surveillance systems	Studies reporting on non-influenza, SARS-CoV-2, and RSV surveillance systems
Studies reporting on whether surveillance systems capture surveillance data, burden of disease data, or vaccine effectiveness data	Studies reporting on aspects of surveillance systems unrelated to data capture, the burden of disease, or vaccine effectiveness (*e.g.* governance, funding)

ICD – international classification of diseases, RSV – respiratory syncytial virus, SARS-CoV-2 – severe acute respiratory syndrome coronavirus two

Data extraction was done by two independent reviewers from a group of nine reviewers (EAM, NA, MN, CS, AGW, EB, UDS, PK, and LV) using a standardised data extraction form. The data extraction form was developed, piloted and appropriately refined using five included studies. Data items extracted from eligible full-text articles included: author (year), location, study aim and design, population under study, syndrome(s) under study (ILI/SARI/acute respiratory infection (ARI)), specific disease under study (influenza/RSV/COVID-19), name, objective and setting of the surveillance system (surveillance/burden of disease/both), ICD code(s) used, implications of surveillance/future recommendations; and assessment/adjustment for potential bias/confounders. Two independent reviewers from a group of nine reviewers (EAM, NA, MN, CS, AGW, EB, UDS, PK, and LV) then performed quality assessment of the included studies using the Joanna Briggs Institute Critical Appraisal tools for prevalence, cohort, and case series studies. All nine reviewers were trained on the procedures and use of the standardised data extraction and quality assessment forms/tools. Any disagreement was resolved through discussions. Meta-analysis was not possible due to the high degree of heterogeneity among studies; therefore, we conducted a narrative synthesis of the study results.

## RESULTS

### Search results

**Figure 1:**
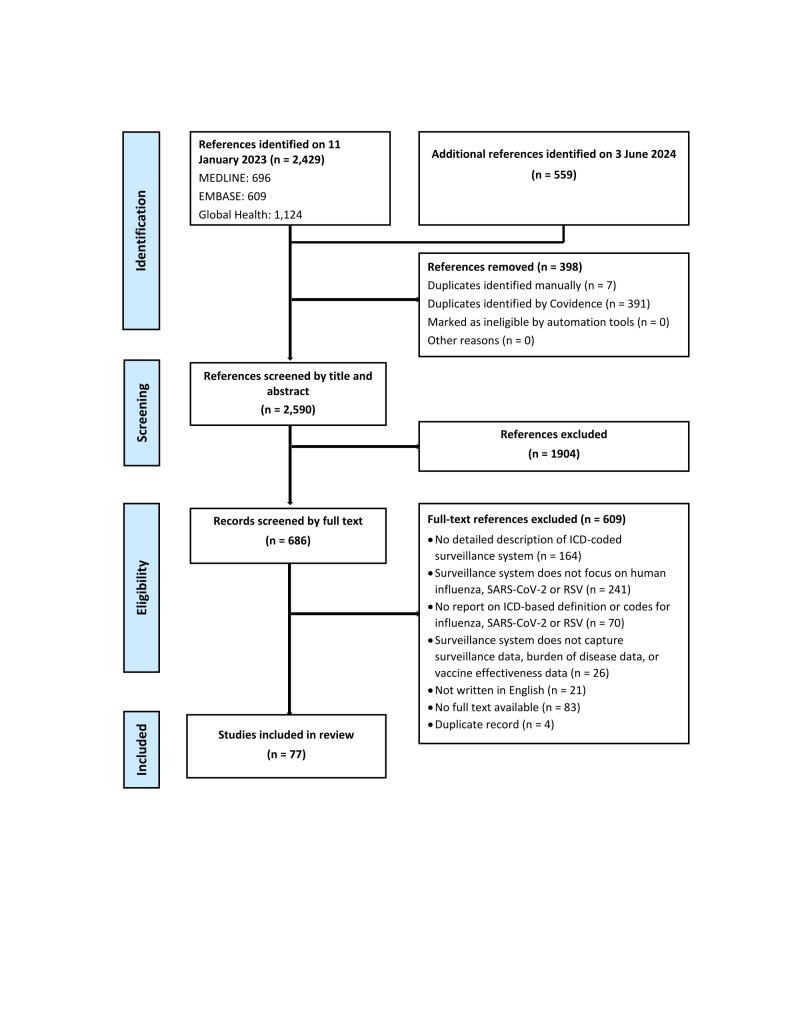
PRISMA flowchart showing the selection of relevant literature.

Through database searches, we identified 2988 records published between 2007–24. After duplicate removal and screening of abstracts and titles, two reviewers independently selected 686 studies for full text screening, utilising the predefined eligibility criteria. Full-text screening resulted in the exclusion of 609 studies owing to the following: no detailed description of ICD-coded surveillance system (n = 164), surveillance system does not focus on human influenza, SARS-CoV-2, or RSV (n = 241), no report on ICD-based definition or codes for influenza, SARS-CoV-2 or RSV (n = 70), surveillance system does not capture surveillance data, burden of disease data, or vaccine effectiveness data (n = 26), not written in English (n = 21), no full text available (n = 83), or duplicate study (n = 4). We included 77 papers in this review [10–86] (**Figure 1**).

### Characteristics of included studies

The included studies were undertaken within 23 countries located across five WHO regions – 32 studies were conducted in the European Region, 32 in the Americas, 10 in the Western Pacific, two in the Eastern Mediterranean, and one in the African Region. Notably, no studies were identified from the South-East Asia Region. Of the papers included, 65 were cross-sectional/observational case studies, 11 were cohort studies, and one was a case series study (Table S1 in the **Online Supplementary Document**).

### Quality assessment

For the cross-sectional/observational case studies, 34 were rated as high quality and 31 as moderate quality. For the cohort studies included, seven were rated as high quality, three as moderate quality, and one as low quality. The case series study included in this review was rated as high quality (Table S2 in the **Online Supplementary Document**).

### Characteristics of ICD-coded surveillance systems and objectives

The 77 included studies reported on 71 surveillance systems. Of these, 35 studies reported on 33 systems recording surveillance data only, 17 studies on 15 systems recording burden of disease data only, and 25 studies on 23 systems recording both surveillance and burden of disease data. Notably, none of these surveillance systems captured vaccine effectiveness data. Surveillance systems had been implemented within the public health care settings only (n = 53), private health care settings only (n = 3), or both public and private health care settings (n = 15). All surveillance systems utilised two major ICD codes (and their clinical modifications). Further, 53 systems utilised ICD-10 codes (75%), 16 systems utilised ICD-9 codes (22%), and two systems utilised both ICD-9 and ICD-10 codes (3%) (Table S3 in the **Online Supplementary Document**).

### ICD codes used for diagnosis of ILI, SARI, ARI, RSV, influenza, or COVID-19

#### Burden of disease

Of the 17 studies reporting on 15 different systems recording burden of disease data, most systems (n = 13, 86%) utilised ICD-10 codes only, one (7%) utilised ICD-9 codes only, and one (7%) utilised both ICD-9 and ICD-10 codes.

For SARI syndromic surveillance, Abdel-Hady et al. [10] reported the use of ICD-10 codes J09–J18, whereas ICD-10 codes B97.8, H66.90, J00, J01, J06, J09–12, J18, J20, J22, J40, and R05 were frequently reported for ILI/SARI syndromic surveillance [50,64,80,81]. These systems were targeted at estimating the incidence and economic burden of hospital admissions and mortality associated with influenza across a population of all ages, using hospital discharge records. For disease-specific surveillance, the most frequently used ICD-10 codes for influenza across systems were J09 and/or J10 (and their sub-codes) [10,15,41,50,61,64,74,80,81], code U07.1 for COVID-19 [36,60,68,69], codes U09.9 and B94.8 for long COVID [36,82], and the codes B97.4, J12.1, J20.5 and J21.0 for RSV [23,41,74,76]. These ICD-10-based codes were extracted from databases, registries, laboratory testing platforms, and disease-specific data models.

The systems which utilised ICD-9 codes focused on estimating the burden of influenza- and RSV-attributable hospitalisations and deaths across a population of all ages, using a disease-specific data model or hospital discharge data and death certificates. Pumarola et al. [64] reported the use of ICD-9 codes 390–459, 480–488, and 460–519 for ILI/SARI syndromic surveillance. However, ICD-9 code 488 was mostly used for influenza-specific surveillance [52,64] while ICD-9 codes 466.11 and 480.1 were utilised for RSV-specific surveillance [52]. These ICD-10-based codes were used in disease-specific data models.

#### Surveillance

Of the 35 studies which described 33 systems recording surveillance data, 22 (67%) of these systems used ICD-10 codes only, 10 (30%) used ICD-9 codes only, and one (3%) used both ICD-9 and ICD-10 codes.

Concerning the use of ICD-10 codes, the most frequently used ICD codes across ILI syndromic surveillance systems were J06, J09–J18, J20–J22, and R50.9 [22,24,58,66,73], whereas the codes J09–J22, U07.1, and U07.2 were mostly used for SARI syndromic surveillance [22,26,28,37,47,77,78,83]. ILI and SARI cases were identified via patients’ emergency department (ED) records using ICD-10 diagnosis codes. For ARI syndromic surveillance, Boender et al. [22] and Cai et al. [27] mainly reported the use of codes J06–J22, J44.0, and B34.9, linked to ED data. Regarding disease-specific surveillance, the most frequently used ICD-10 codes for influenza across systems were J09 and/or J10 (and their sub-codes) [13,24,26,28,29,37,54,58,73,78,83], code U07.1 for COVID-19 [22,31,32,44,47,55,77,78,83,84], and the codes B97.4, J12.1, J20.5 and J21.0 for RSV [20–22,26–28]. These ICD-10-based codes were extracted from databases, registries, laboratory testing platforms, sentinel electronic data collection, patients’ medical records, and ED data.

Most studies reported the use of ICD-9 codes 460–466 and 480–487 (and their sub-codes), 780.6, and 786.2 in ILI syndromic surveillance systems [17,33,35,51,63,71,79], while codes 390–459, 480–488, and 460–519 were utilised for ILI/SARI/ARI syndromic surveillance [19,67]. These ICD-9 codes were mainly linked to patients’ medical records. For disease-specific surveillance, ICD-9 code 487.1 was frequently used for influenza-specific surveillance [19,29,33,35,63,67], while ICD-9 codes 079.6, 466.11, and 480.1 were utilised for RSV-specific surveillance [30,48,51]. These ICD-9-based codes were obtained from databases and sentinel laboratories.

#### Burden of disease and surveillance

Of the included studies, 25 examined 23 systems recording both surveillance and burden of disease data. Most systems (n = 18, 78%) utilised ICD-10 codes, while five (22%) used ICD-9 codes.

The commonly reported SARI syndromic surveillance ICD-10 codes were J09–J18, and J20–J22 [18,34,65,75,85,86]. The codes J09–J18 and R06 were used for ILI surveillance [14,39] and the codes J09–J18 and J20–J22 for ARI syndromic surveillance [46,57,59], while codes J09–J12, J20, and J21 were reported for ILI/SARI/ARI syndromic surveillance [11,46]. ILI, ARI and SARI cases were identified via patients’ ED records, hospital discharge records and/or clinical databases. For disease-specific surveillance, the most frequently used ICD-10 codes for influenza across systems were J09 and J10 [11,14,18,34,39,42,46,53,57,65,75,86], codes U07.1 and J12.82 for COVID-19 [40,43,56,72], and the codes J12.1, J20.5, and J21.0 for RSV [11,49,57,85]. These ICD-10-based codes were extracted from databases, registries, laboratory testing platforms, sentinel sites data, and disease-specific data models.

Regarding systems which utilised ICD-9 codes, Amodio et al. [12] and Keck et al. [45] reported the use of codes 480 and 487 for ILI surveillance, whereas Ortiz et al. [62] reported the codes 96.7, 460–466, 480–486, 518, 995.92, and 785.52 for SARI surveillance. These ICD-9-based codes were extracted from hospital discharge records, databases, and ambulatory visits and hospitalisations. However, ICD-9 codes 487 and 488 were mostly used for influenza-specific surveillance [45,62,70], while codes 043, 480.4, 518.9, and 519.7 were utilised for COVID-19-specific surveillance [38]. These ICD-9-codes were obtained from hospital discharge records, registry and database.

### Sensitivity, specificity, positive predictive value, and negative predictive value of ICD codes within surveillance systems

Among the included studies, 17 [11,13,14,19,27,29,32,33,35,43,55,60,61,65,66,72,74] provided reports on the statistical estimates (sensitivity, specificity, positive predictive value (PPV), and negative predictive value (NPV)) for influenza, SARS-CoV-2, or RSV-specific ICD codes utilised within surveillance systems (**Table 2**). No meta-analysis was conducted due to the small number and heterogeneity of the studies included.

**Table 2:** Sensitivity, specificity, positive predictive value, negative predictive value, and limitations of using ICD-coded data within surveillance systems

Study	Location	Name of system	Correlation of ICD codes with laboratory-confirmed diagnosis	Sensitivity, specificity, PPV, and NPV of ICD codes used	Limitations of the use of ICD-coded data within disease-specific surveillance systems
Abdel-Hady et al., 2018 [10]	Oman	Nabdh Al Shifa (computerised online system)	No	N/A	N/A
Alchikh et al., 2019 [11]	Germany	Quality management programme at a Berlin children's hospital with the Robert Koch Institute	Yes	ICD-10 diagnoses relative to viral (mono-)infections with influenza virus: sensitivity = 38.84% (95% CI = 34.88–42.92), specificity = 97.98% (95% CI = 97.53–98.36). ICD-10 diagnoses relative viral (mono-)infections with RSV: sensitivity = 49.63% (95% CI = 46.11–53.14), specificity = 96.41% (95% CI = 95.82–96.93)	Physician bias, lack of familiarity, time constraints, coding errors
Amodio et al., 2014 [12]	Italy	The Regional Hospital Discharge Database, WHO FluNet database	No	N/A	Underestimation of infections
Antoon et al., 2024 [13]	USA	NVSN	Yes	ICD-10 influenza diagnoses in the ED: sensitivity = 48.6% (95% CI = 47.6–49.5), specificity = 98.0% (95% CI = 97.8–98.3), PPV = 88.6% (95% CI = 88.0–89.2), NPV = 85.9% (95% CI = 85.3–86.6). ICD-10 influenza diagnoses among inpatients: sensitivity = 70.7% (95% CI = 69.8–71.5), specificity = 98.2% (95% CI = 98.0–98.5), PPV = 82.8% (95% CI = 82.1–83.5), NPV = 96.5% (95% CI = 96.2–96.9)	Underestimation of infections
Aysert-Yildiz et al., 2019 [14]	Turkey	GIHSN	Yes	Hospital admission ICD-10 codes: sensitivity = 6.6%, specificity = 98.9%. Hospital discharge ICD-10 codes: sensitivity = 6.6%, specificity = 94.7%	Underestimation of infections
Azziz-Baumgartner et al., 2013 [15]	Argentina	PAHO’s mortality databases and Argentina’s hospitalization databases	No	N/A	N/A
Bagarella et al., 2022 [16]	Italy	Structured and unstructured data from visits to three EDs of a local health assessment unit in Lombardy	No	N/A	N/A
Bellazzini et al., 2011 [17]	USA	ED syndromic surveillance system	No	N/A	N/A
Bernadou et al., 2023 [18]	France	PMSI	No	N/A	N/A
Betancourt et al., 2007 [19]	USA	ESSENCE	No	ESSENCE algorithms (syndromes based on lists of selected ICD-9 codes) for detecting respiratory disease: sensitivity = 65.7%, specificity = 95.6%, PPV = 81.3%, NPV = 90.4%	Lack of familiarity, time constraints
Bhatt et al., 2023 [20]	USA	2019 HCUP-KID	No	N/A	Coding errors
Blatt et al., 2024 [21]	USA	EHR data from four health care systems: SUNY Health System, Duke University Health System, Renown Children's Hospital, and Tampa General Hospital/ University of South Florida	No	N/A	N/A
Boender et al., 2022 [22]	Germany	The AKTIN Emergency Department Data Registry and the ESEG project	No	N/A	Underestimation of infections
Bouckaert et al., 2023 [23]	Belgium	B-HDDS	No	N/A	Underestimation of infections
Bouzille et al., 2018 [24]	France	eHOP clinical data warehouse technology of the academic hospital of Rennes	No	N/A	N/A
Bruzda et al., 2021 [25]	USA, Dominican Republic, El Salvador, Honduras	CREDO system	No	N/A	N/A
Buda et al., 2017 [26]	Germany	Hospitals belonging to the HELIOS Kliniken Gmb	No	N/A	N/A
Cai et al., 2020 [27]	Germany	SEEDARE, ICOSARI, and from the virological surveillance at the Robert Koch Institute	Yes	RSV-specific ICD-10 codes (J12.1, J20.5, J21.0): sensitivity = 6% (95% CI = 3–12), specificity = 99.8% (95% CI = 99.6–99.9)	Underestimation of infections, physician bias
Cai et al., 2020 [28]	Germany	ICOSARI database	No	N/A	Underestimation of infections, physician bias
Chow et al., 2020 [29]	USA	The US FluSurv-NET	Yes	ICD-9-CM and ICD-10 discharge codes for influenza and related diagnosis: influenza code (ICD-9-CM codes 487–488, ICD-10 codes J09–J11): sensitivity = 82.0%. Influenza and pneumonia code (ICD-9-CM codes 480–488, ICD-10 codes J09–J18): sensitivity = 86.0%. Respiratory and circulatory code (ICD-9-CM codes 460–519 and 390–459, ICD-10 codes J00–J99 and I00–I99): sensitivity = 99.0%	Underestimation of infections, coding errors
Cocchio et al., 2023 [30]	Italy	Hospital discharge records of Veneto's database	No	N/A	Testing practices may not be generalisable
Cocoros et al., 2023 [31]	USA	ESP public health surveillance platform	No	N/A	Underestimation of infections
Eick-Cost et al., 2022 [32]	USA	DMSS	Yes	ICD-10-CM (U07.1) for COVID-19: sensitivity = 29–66%, specificity = 99%, PPV = 94%. ICD-10-CM codes for COVID-like illness: Sensitivity = 76–79%, specificity = 65–86%, PPV = 15–62%	Underestimation of infections
Elkin et al., 2016 [33]	USA	Biosense biosurveillance system	Yes	ICD-9 codes for Influenza: sensitivity = 45.6%, specificity = 97.9%, PPV = 66.2%, NPV = 95.1%	Underestimation of infections
Farah et al., 2023 [34]	Lebanon	SARI sentinel surveillance system, and the Ministry of Public Health’s hospital billing database	No	N/A	Underestimation of infections
Feemster et al., 2016 [35]	USA	EHRs from a large paediatric network of clinics	No	ICD-9-CM diagnosis codes compared to chart review: sensitivity = 99.2% (95% CI = 98.1–100), specificity = 98.1% (95% CI = 97.2–98.9), PPV = 95.1% (95% CI = 92.8–97.4), NPV = 99.7% (95% CI = 93.3–100). ICD-9-CM diagnosis codes compared to parental interview: sensitivity = 3.7% (95% CI = 2.4–4.9), specificity = 99.7% (95% CI = 99.7–99.8), PPV = 50.0% (95% CI = 44.9–55.1), NPV = 94.9% (95% CI = 93.3–96.6).	Underestimation of infections
Fung et al., 2023 [36]	USA	CMS Virtual Research Data Centre	No	N/A	Underestimation of infections
Gerbier-Colomban et al., 2014 [37]	France	UrgIndex surveillance system at the North Hospital Group of the Lyon University Hospitals	No	N/A	N/A
Giordani et al., 2024 [38]	Italy	Hospital discharge records (collected by Italian MoH), linked with National Tax Registry	No	N/A	Underestimation of infections
Girit et al., 2017 [39]	Turkey	GIHSN	No	N/A	N/A
Gundlapalli et al., 2021 [40]	USA	Death certificates from CDC	No	N/A	N/A
Habbous et al., 2023 [41]	Canada	DAD and NACRS	No	N/A	Underestimation of infections
Hagiwara et al., 2022 [42]	Japan	RWD database	No	N/A	N/A
Ishiguro et al., 2024 [43]	Japan	Claims data from the National Center for Global Health and Medicine Hospital	No	ICD-10 diagnosis code-only algorithm for hospitalization with COVID-19: sensitivity = 92.6% (95% CI = 91.0–94.0), specificity = 88.1% (95% CI = 87.9–88.3), PPV = 11.0% (95% CI = 10.4–11.7), NPV = 99.9% (95% CI = 99.9–99.9).	N/A
Johnson et al., 2022 [44]	USA	Biobank at CCPM	No	N/A	N/A
Keck et al., 2014 [45]	USA	The Indian Health Service’s Influenza Awareness System	No	N/A	N/A
Khanh et al., 2021 [46]	Vietnam	Vietnam's SARI sentinel surveillance system, Vietnam's MoH EMRs system	No	N/A	N/A
Leiner et al., 2023 [47]	Germany	IQM network	No	N/A	N/A
Light et al., 2008 [48]	USA	Florida Department of Health's epidemiology surveillance network	Yes. A positive correlation between RSV test data and hospitalisations	N/A	Underestimation of infections
Loubet et al., 2024 [49]	France	PMSI	No	N/A	Underestimation of infections, physician bias
Mad Tahir et al., 2023 [50]	Malaysia	MY-DRG Casemix database (of a teaching hospital in Malaysia)	No	N/A	N/A
Marsden-Haug et al., 2007 [51]	USA	An automated ILI surveillance report incorporated into ESSENCE	Yes. ICD-9 codes within ESSENCE ILI group were associated with specimens positive for respiratory pathogens, including influenza	N/A	N/A
Matias et al., 2017 [52]	USA	Hospitalisation data from US Nationwide Inpatient Sample, and virological data from FluView	Yes. Statistical modelling approach included time series of influenza and RSV laboratory-confirmed patterns for hospitalization	N/A	Overestimation of risk, physician bias, coding errors
Mattiuzzi et al., 2023 [53]	USA	CDC WONDER Online Database	No	N/A	N/A
McLeod et al., 2009 [54]	New Zealand	Wellington ED Respiratory Syndromic Surveillance System	No	N/A	N/A
McMurry et al., 2024 [55]	USA	An open-source AI–based natural language processing pipeline that includes a large language model	Yes	ICD-10 code-based COVID-19 symptom monitoring: sensitivity = 30%, specificity = 99.4%, PPV = 90.6%	Underestimation of infections
Milliren et al., 2023 [56]	USA	Paediatric Hospital Information System	No	N/A	Coding errors
Mira-Iglesias et al., 2022 [57]	Spain	VAHNSI	Yes. Laboratory-confirmed RSV results were used to determine RSV positivity rates according to ICD-10 discharge diagnoses for RSV-associated disease	N/A	N/A
Moore et al., 2011 [58]	Australia	SynSurv	Yes. Five ICD-10 codes correlated with laboratory confirmed influenza cases (J06, J11, J22, B34, J18)	N/A	Physician bias
Motlogeloa et al., 2023 [59]	South Africa	Data from the South African Medical Research Council Respiratory and Meningeal Pathogens Research Unit database, and the Discover Medical Insurance Scheme claims database	No	N/A	N/A
Moura et al., 2024 [60]	Canada	CCEDRRN registry linked to administrative diagnostic codes	Yes	Performance of ICD-10 code (U07.1), with PCR testing as the reference standard. Hospitalised patients sensitivity = 93.6% (95% CI = 93.0–94.1%), specificity = 99.8% (95% CI = 99.7–99.8), PPV = 98.6% (95% CI = 98.4–98.9), NPV = 98.8% (95% CI = 98.7–98.9). ED patients sensitivity = 83.0% (95% CI = 82.1–83.9), specificity = 97.5% (95% CI = 97.3–97.7%), PPV = 90.1% (95% CI = 89.4–90.8%), NPV = 95.5% (95% CI = 95.2–95.7)	Underestimation of infections
Murray et al., 2023 [61]	Australia	NATA approved, batch testing, in-house real-time PCR platform	Yes	ICD-10 coding (J09, J10) of inpatients with laboratory-confirmed influenza: sensitivity = 76.4%	Underestimation of infections
Ortiz et al., 2014 [62]	USA	State Inpatient Databases from Arizona, California, and Washington, and Regional influenza surveillance data from CDC	No	N/A	Underestimation of infections
Pattie et al., 2009 [63]	USA	AHLTA	No	N/A	N/A
Pumarola et al., 2023 [64]	Spain	PHDB for Spain	No	N/A	Coding errors, underestimation of infections, coding practices may not be generalisable
Ramos-Rincon et al., 2024 [65]	Spain	SNSSHD, specifically the Hospital Care Activity Record - Minimum Basic Data Set (Registro de actividades especializadas-Conjunto Mínimo Básico de datos)	Yes	ICD-10 codes (J09, J10, J11): sensitivity = 79.87%, specificity = 99.72%, PPV = 86.71%, NPV = 99.54%	Underestimation of mortality, coding errors, overestimation of metrics such as length of hospital stay, admission to ICU), mortality rates, and associated costs
Ramsay et al., 2010 [66]	Australia	Community-based Victorian GP surveillance program	Yes	ICD-10 syndromic codes for ILI: among admitted patients sensitivity = 43%. Among discharged patients sensitivity = 40%	Underestimation of infections
Reed et al., 2014 [67]	USA	EIP Influenza Surveillance Network	No	N/A	Underestimation of infections, physician bias
Ricoca Peixoto et al., 2023 [68]	Portugal	Clinical registries of Portuguese NHS hospitals	No	N/A	Underestimation of infections
Saleh et al., 2024 [69]	USA	IDR i2b2 platform	No	N/A	N/A
San-Roman-Montero et al., 2019 [70]	Spain	National Surveillance System for Hospital Data (Conjunto Mínimo Básico de Datos)	No	N/A	N/A
Schirmer et al., 2010 [71]	USA	ESSENCE	No	N/A	Underestimation of infection, coding errors
Shappell et al., 2023 [72]	USA	Mass General Brigham Healthcare System	Yes	ICD-10 discharge diagnosis code for COVID-19 (U07.1 or J12.82): sensitivity = 98.4% (95% CI = 91.3–100), specificity = 39.5% (95% CI = 24.0–56.6), PPV = 72.6% (95% CI = 61.8–81.8), NPV = 93.8% (95% CI = 69.8–99.8)	Underestimation of infections
Sigmundsdottir et al., 2010 [73]	Iceland	CHS-CDC	No	N/A	Physician bias
Sivakumaran et al., 2023 [74]	Wales	SAIL Databank	Yes	ICD-10 diagnosis codes (J09, J10, J11) used to identify influenza-associated COPD admissions: sensitivity = 59.2% (95% CI = 52.9–65.1), specificity = 97.4% (95% CI = 96.2–98.3), PPV = 86.1% (95% CI = 80.6–90.2), NPV = 89.7% (95% CI = 88.3–91.0). ICD-10 diagnosis codes (J12.1, J20.5, J21.0, B97.4) used to identify RSV-associated COPD admissions: sensitivity = 12.1% (95% CI = 5.9–21.0), specificity = 100% (95% CI = 99.7–100), PPV = 100, NPV = 93.9% (95% CI = 93.5–94.4)	Underestimation of infections
Sotomayor et al., 2018 [75]	Chile	SARI sentinel surveillance database, Records of hospital discharges and national deaths, managed by the DSHI of the MoH of Chile	No	N/A	Underestimation of mortality rates
Taylor et al., 2016 [76]	UK	CPRD, HES database, ONS database, UK national surveillance system at PHE	No	N/A	Changes in coding practices
Thiam et al., 2022 [77]	France	SurSaUD®	No	N/A	Underestimation of infections, coding errors
Torres et al., 2023 [78]	Portugal	SARI sentinel surveillance system	No	N/A	Underestimation of infections
Trucchi et al., 2019 [79]	Italy	Syndromic Surveillance System	No	N/A	N/A
Wan Puteh et al., 2023 [80]	Malaysia	Casemix system	No	N/A	Coding practice may not be generalisable
Wan Puteh et al., 2024 [81]	Malaysia	Casemix system	No	N/A	Coding practice may not be generalisable
Wander et al., 2023 [82]	USA	US Department of VA’s Corporate Data Warehouse and CSDR	No	N/A	Coding errors
Wells et al., 2022 [83]	Scotland	SARI surveillance system: using data from SMR01 and RAPID	No	N/A	Coding errors
Whittaker et al., 2023 [84]	Norway	NPR and NoPaR	No	N/A	Physician bias, coding errors
Wick et al., 2023 [85]	Germany	German Institute for the Hospital Remuneration System	No	N/A	Underestimation of infections
Widgren et al., 2010 [86]	Denmark	Danish National Patient Registry	No	N/A	Underestimation of infections

AI – artificial intelligence, AHLTA – armed forces health longitudinal technological application, ARI – acute respiratory infection, B-HDDS – Belgian hospital discharge data set, CCEDRRN – Canadian COVID-19 emergency department rapid response network, CCPM – Colorado centre for personalized medicine, CDC – Centre for Disease Control and Prevention, CHS-CDC – Centre for Health Security and Communicable Disease Control, CI  – confidence interval, CMS – Centres for Medicare and Medicaid Services, CPRD – clinical practice research datalink, CREDO – clinical rotation evaluation and documentation organiser, CSDR – COVID-19 shared data resource, DAD – discharge abstract database, DMSS – defense medical surveillance system, DSHI – department of statistics and health information, ED – emergency department, EHR – electronic health record, EIP – emerging infections program, ESEG – Erkennung und Steuerung Epidemischer Gefahrenlagen, ESP – electronic medical record support for public health, ESSENCE – Electronic Surveillance System for the Early Notification of Community-based Epidemics, FluSurv-NET – Influenza Hospitalisation Surveillance Network, GIHSN – Global Influenza Hospital Surveillance Network, GP – general practitioner, HCUP-KID – health care cost and utilisation project’s kids inpatient database, HES – hospital episode statistics, ICOSARI – ICD-10 code-based surveillance system for severe acute respiratory infections, IDR – integrated data repository, ILI – influenza-like illness, IQM – initiative of quality medicine, MoH – ministry of health, MY-DRG – Malaysian Diagnosis Related Group, NACRS – national ambulatory care reporting system, NATA – National Association of Testing Authorities, NHS – National Health Services, NoPaR – Norwegian Pandemic Registry, NPR – Norwegian Patient Registry, NVSN – new vaccine surveillance network, N/A – not available, ONS – Office of National Statistics, PAHO – Pan American Health Organization, PCR – polymerase chain reaction; PHDB – projected hospitalisations database, PHE – Public Health England, PMSI – Programme de M´edicalisation des Systemes d’information, RAPID – rapid preliminary inpatient data set, RWD – real-world data, SAIL – secure anonymized information linkage, SARI – severe acute respiratory infections, SEEDARE – ICD-10-based influenza and other ARI surveillance systems sentinel electronic data collection system, SMR01 – the general acute inpatient and day case, SNSSHD – Spanish National Surveillance System for Hospital Data, SUNY – State University of New York, SurSaUD – Surveillance Sanitaire des Urgences et des Décès, SynSurv – Victorian Syndromic Surveillance, VA – Veterans Affairs, VAHNSI – Valencia Hospital Network for the Study of Influenza, WHO – World Health Organization

#### ICD-10 codes

Of the included studies, five studies addressed hospital admissions data for influenza-specific surveillance [13,14,61,65,74]. In these studies, low-to-moderate sensitivity (6.60–79.87%), high specificity (97.40–99.72%), moderate PPV (82.80–86.71%) and high NPV (89.70–99.54%) were reported for influenza-specific ICD-10 codes (mainly J09 and J10). Among these, two studies [11,13] reported low sensitivity (38.84–48.6%) and high specificity (97.98–98.00%) for influenza-specific ICD-10 codes from ED data. However, Ramsay et al. [66] reported low sensitivities of 43.00% and 40.00% among admitted patients and discharged patients, respectively for ICD-10-coded ILI syndromic surveillance.

High sensitivity (92.60–98.40%), low-to-high specificity (39.50–99.80%), low-to-high PPV (11.00–98.60%), and high NPV (93.80–99.90%) for COVID-19-specific ICD-10 code (U07.1) were documented in three studies [43,60,72] which used hospital admissions data, while two studies [55,60] showed low-to-moderate sensitivity (30.00–83.00%), high specificity (97.50–99.40%) and high PPV (90.10–90.60%) for COVID-19-specific ICD-10 code (U07.1) from ED data.

Further, two studies [27,74] reported low-to-high sensitivity (6.00–99.80%) and low-to-high specificity (12.10–100.00%) for RSV-specific ICD-10 codes (J12.1, J20.5, and J21.0), using hospital admissions data. However, Alchikh et al. [11] reported a low sensitivity (49.63%) and high specificity (96.41%) for RSV-specific ICD-10 codes (J12.1, J20.5, and J21.0) from ED data.

#### ICD-9 codes

Only Elkin et al. [33] reported low sensitivity (45.60%), high specificity (97.90%), low PPV (66.20%) and high NPV (95.10%) for influenza-specific ICD-9 code 487.1, using both hospital admissions and ED data. Betancourt et al. [19] documented a low sensitivity (65.70%) and high specificity (95.60%) for ILI ICD-9 diagnosis using both hospital admissions and ED data, while Feemster et al. [35] reported a high sensitivity (99.20%) and high specificity (98.10%) using data from electronic health records.

#### Both ICD-9 and ICD-10 codes

Chow et al. [29] solely reported the sensitivities for the use of both ICD-9 and ICD-10 discharge codes for influenza and related diagnoses. Influenza code (ICD-9-clinical modification codes 487–488, and ICD-10 codes J09–J11) had a moderate sensitivity (82.00%), whereas influenza and pneumonia code (ICD-9-clinical modification codes 480–488, and ICD-10 codes J09–J18) had a moderate sensitivity (86.00%).

### Limitations of ICD-coded data within surveillance systems

Among included studies, 49 described the limitations of using ICD-coded data within influenza, SARS-CoV-2, and RSV surveillance systems (**Table 2**; Table S4 in the **Online Supplementary Document**). Most of these studies reported underestimation of infection or mortality rates as the main limitation of using ICD codes within these syndromic or disease-specific surveillance systems [12–14,22,23,27–29,31–36,38,41,48,49,55,60–62,64–68,71,72,74,75,77,78,85,86]. The usefulness of ICD codes for surveillance purposes was also limited by other factors, such as coding errors [11,20,29,52,56,64,65,71,77,82–84], physician bias [11,27,28,49,52,58,67,73,84], coding practices may not be generalisable [64,80,81], lack of familiarity [11,19], time constraints [11,19], overestimation of risk [52,65], testing practices may not be generalisable [30], and changes in coding practices [76].

### Adjustment for potential bias and confounders

Of the included studies, 29 [11,13,15,16,18,28,34,36,38,41,42,46,47,49,50,52,55,57,60-62,64,65,67,68,70,76,82,86] adjusted for potential confounders, mainly age, gender, seasonality, and comorbidities. Of these, five studies [38,50,62,68,76] clearly reported on adjusting for potential bias.

## DISCUSSION

In this systematic review, we included 77 eligible studies from 23 countries located across five WHO regions, providing evidence on the key characteristics of influenza, SARS-CoV-2, and RSV surveillance systems that utilise ICD-coded data and assess their performance. Overall, 35 studies reported on 33 systems recording surveillance data only, 17 studies reported on 15 systems recording burden of disease data only, and 25 studies reported on 23 systems recording both surveillance and burden of disease data. Most surveillance systems have been implemented within public health care settings. All surveillance systems utilized ICD-10 and/or ICD-9 codes. For disease-specific surveillance, the most frequently used ICD-10 codes for influenza across systems were J09 and/or J10, code U07.1 for COVID-19, and the codes B97.4, J12.1, J20.5, and J21.0 for RSV. However, ICD-9 codes 487 and 488 were mostly used for influenza-specific surveillance, and codes 466.11 and 480.1 were utilised for RSV-specific surveillance. The performance of these ICD codes within surveillance systems remains inconclusive. ICD-10 codes generally showed a low-to-moderate sensitivity and high specificity for influenza diagnosis, low-to-high sensitivity and specificity for COVID-19 diagnosis, and low-to-high sensitivity and specificity for RSV diagnosis, using hospital admissions or ED data. Conversely, ICD-9 codes showed low sensitivity and high specificity for influenza diagnosis using hospital admissions or ED data. The usefulness of these ICD codes for surveillance purposes was mainly limited by their underestimation of infection and/or mortality rates for influenza, COVID-19 or RSV.

ICD-10-code-based surveillance systems in our review captured data on codes used for COVID-19 diagnosis, as opposed to ICD-9-code-based systems, when data sources are from hospital admissions or ED. ICD-10 codes had been implemented on 1 October 2015, prior to the introduction of COVID-19 globally, and could possibly account for this disparity. Concerning sensitivity, specificity, PPV and NPV, the outcomes for each of these included studies appear to differ as a result of variations in the definition of primary and secondary diagnosis in these ICD-coded health data, correlation of ICD codes with laboratory-confirmed diagnosis across settings, objective and setting of surveillance system, use of hospital admissions or ED data, population under study, and/or geographic region.

The sensitivity and specificity of ICD-code-based case definitions for influenza, utilising hospital admissions data, are consistent with results from the systematic review by Hanquet et al. [87], which showed that most ICD code-based algorithms had low sensitivity and high specificity for hospitalised cases of lower tract respiratory infections in adults, except for COVID-19-related lower tract respiratory infections. For influenza-specific surveillance, ICD-10 codes J09 and/or J10 (and their subcodes) and ICD-9 codes 487 and 488 were most frequently used in included papers. The use of these codes within surveillance systems is consistent with the WHO’s sentinel surveillance classification for ILI and SARI cases [88]. However, the accuracy of these codes for identifying influenza in hospital admissions or EDs remains inconclusive in our review. Also, the determination of the ICD codes that would enhance the sensitivity of ICD code-based case definitions in influenza surveillance systems is unclear. The use of a combination of these ICD codes, with laboratory testing, may enhance the identification of influenza cases in surveillance systems [6].

ICD-10-code-based identification (U07.1) was commonly used across COVID-19-related surveillance systems in our included studies and generally demonstrated high sensitivity in identifying hospitalised COVID-19 patients, though inconclusive. This is in line with the findings from other studies [89,90] which reported the use of code U07.1 in identifying hospitalised COVID-19 cases, although the performance of U07.1 may vary across periods and populations. Our results suggest that code U07.1 could be paired with condition-defining diagnoses (such as influenza or pneumonia) for surveillance on hospitalised COVID-19 patients with incomplete diagnostic laboratory results.

The findings of our review show that RSV-specific ICD-10 codes (B97.4, J12.1, J20.5 and J21.0) were less sensitive and specific in detecting laboratory-confirmed RSV infections, using hospital admission or ED data. This suggests that algorithms employed in RSV surveillance systems for admitted or ED patients with RSV may be ineffective when laboratory tests are not available. Generally, RSV surveillance is a consequence of SARI or ILI surveillance [91,92]. The capacities of existing surveillance systems for RSV may be affected by the testing strategy for influenza. To address this challenge, ICD-10-code-based hospital admissions or ED data with laboratory-confirmed RSV tests could be a useful strategy in enhancing the accuracy of RSV-specific ICD-10 codes used in the detection of true RSV infections [93]. This would enhance the roll-out of the recently licensed RSV vaccine or passive immunization products targeted at reducing RSV disease burden across multiple (mainly high-income) countries [94].

The usefulness of these ICD codes for surveillance purposes was mainly limited by their underestimation and misclassification of infection and/or mortality rates for influenza, COVID-19 or RSV. These findings are in accordance with that of Hayward et al. [95] who found that a surveillance system using ICD-10 codes underestimated and misclassified patients with non-alcoholic fatty liver diseases. Kurbasic et al. [96] and Burles et al. [97] also cited underestimation and coding errors as limitations of using ICD codes. There could be several factors which may influence the accuracy of influenza, SARS-CoV-2, and RSV diagnosis coding. For example, the differential utilisation of rapid tests and their differing sensitivity and specificity may contribute to underestimation of infection and/or mortality rates attributable to influenza, COVID-19 or RSV [98]. Also, clinicians’ perception of the circulation of these respiratory viruses, as well as the varying hospital policies across settings or geographic regions, may influence the clinical diagnosis of influenza, SARS-CoV-2, and RSV [58,98]. It is thus imperative that regional and national guidelines should be consulted for more detailed recommendations on influenza, SARS-CoV-2, and RSV diagnosis coding and reporting. For example, the United States’ Centres for Medicare and Medicaid Services and the National Centre for Health Statistics periodically publish the ‘ICD-10-CM Official Guidelines for Coding and Reporting’ [99] to assist clinicians and coders in identifying and ensuring consistent classification of diagnoses of these respiratory diseases. Additionally, the implementation of the new ICD-11 coding system by countries could significantly mitigate these coding challenges through its post coordination system and clearer guidance [100].

Although most high-income countries have communicable disease surveillance systems in place, low- and middle-income countries are faced with major barriers to the surveillance of communicable diseases, as evident from the results of our systematic review. These barriers include, but not limited to, fragmented health care systems with poor data flow, health workforce shortage and inequitable distribution, and lack of or inadequate diagnostic facilities (such as clinical laboratories) [101,102]. Therefore, the adoption of modern technology, education and capacity building for the health workforce, and improving diagnostic capacity and access will enhance the surveillance of influenza, SARS-CoV-2, and RSV in low- and middle-income countries. This can be achieved through WHO’s Integrated Disease Surveillance and Response system in the WHO African Region [103] and the Strategic Framework for Action for Strengthening Surveillance, Risk Assessment and Field Epidemiology for Health Security Threats in the WHO South-East Asia Region [104].

### Strengths and limitations

To our knowledge, this is the first review which focuses on the key characteristics and performance of influenza, SARS-CoV-2 and RSV surveillance systems using ICD-coded data. In addition, this review followed a robust systematic review methodology. First, a protocol was developed, which was adhered to. Comprehensive search strategies were created for the three databases searched. Covidence software was used for screening with two independent reviewers being involved in the screening, data extraction and quality assessment stages.

However, this review has some limitations. We did not include non-English and grey literature/unpublished studies, thereby making this review prone to language and publication bias, respectively. Owing to high heterogeneity, we were only able to conduct a narrative synthesis. Additionally, the choice of quality assessment tools to assess the quality of included studies was challenging due to data complexity and significant heterogeneity in the design and objectives of included studies. Although the authors provided information related to study participants, inadequate information was provided to assess the suitability of the statistical analysis conducted and participants’ response rates, thereby affecting study quality. Furthermore, the included studies spanned a wide range of years (2007–24) – a period which highlights a significant transition from ICD-9 to ICD-10 coding systems. This could impact the comparability of studies conducted within this period due to increased specificity in the use of ICD-10 codes and changes in coding practices.

## CONCLUSIONS

Our review provides valuable insights into the key characteristics and performance of influenza, SARS-CoV-2, and RSV surveillance systems that utilise ICD-coded data. These systems mainly captured syndromic- or disease-specific surveillance data, burden of disease data, or both, utilising ICD-10 and/or ICD-9 codes. None of these surveillance systems captured vaccine effectiveness data. The performance of ICD codes for ILI, ARI, SARI, influenza, SARS-CoV-2, or RSV surveillance remains inconclusive, although using only ICD-coded data within these surveillance systems may underestimate infection and/or mortality rates attributable to influenza, SARS-CoV-2, or RSV. Future studies should focus on the determination of the combination of ICD codes that would improve the sensitivity of ICD code-based case definitions in influenza, SARS-CoV-2, and RSV surveillance systems, as well as gather more data from low- and middle-income countries located within WHO’s African and South-East Asia Regions. For example, the inclusion of all codes related to influenza (ICD-9 codes 487.XX–488.XX and ICD-9 codes J09.XX–J10.XX) may correspond most closely with SARI surveillance. Furthermore, the integration of ICD-coded surveillance of vaccine-preventable diseases into existing systems focused on capturing either surveillance data or burden of disease data – through shared infrastructure for specific components of surveillance such as laboratory systems and data management – would enhance and monitor the quality of surveillance systems and their ability to adapt to new data needs, such as for the effectiveness of new vaccines, as part of WHO’s Immunisation Agenda 2030.

## Additional material


Online Supplementary Document
