# Supplementary material for: Characteristics of influenza, SARS-CoV-2, and RSV surveillance systems that utilise ICD-coded data: a systematic review
Source: J Glob Health. 2025 May 23;15:04177. doi: 10.7189/jogh.15.04177 (PMC12100576; doi:10.7189/jogh.15.04177)
Supplement: Online Supplementary Document [file jogh-15-04177-s001.pdf]

Supplement to: Agyei-Manu E, Atkins N, Nundy M, St-Jean C, Gornall-Wick A, Birley E, De Silva U, Krishan P, Vokey L, Dozier M, McSwiggan E, McQuillan R, Theodoratou E, Shi T. Characteristics of influenza, SARS-CoV-2, and RSV surveillance systems that utilise ICD-coded data: A systematic review. J Glob Health. 2025;15:04177.

Appendix S1: Study Protocol.

Research Question: What are the key characteristics of influenza, SARS-CoV-2, and RSV surveillance systems that utilise ICD-coded data, and how well do they work?

Introduction:

The International Statistical Classification of Diseases and Related Health Problems (ICD) is the global standard for diagnostic health information (ICD, 2022). The ICD operates on a global scale, and it sees the systematic recording, analysis, interpretation and comparison of mortality and morbidity data, highlighting the extent, causes, and consequences of morbidity and mortality (ICD, 2022). On January 1st, 2022, the eleventh, and most recent revision of the ICD (ICD-11) came into effect, but this review will also include articles utilising the previous ICD codes, including ICD-9 and ICD-10 codes (ICD, 2022).

Surveillance systems regularly utilise ICD-coded data, including systems analysing trends of Influenza, SARS-CoV-2, and RSV (Buda *et al,* 2017; Boender *et al,* 2022). ICD-coded data implemented within reporting systems can enable timely, reliable information to be produced regarding respiratory viruses, and this can enable quick, evidence-based decision-making to occur, due to the production and use of reliable data (WHO, 2022; Buda *et al,* 2017).

Key Objectives:

The proposed rapid review aims to describe the key characteristics of influenza, SARS-CoV-2, and RSV surveillance systems that utilise ICD-coded data, or sets of codes used as proxy data, and assess the extent of their performance. This review will do so by characterising included publications in these key aspects:

- Characterise the objectives of these ICD-coded surveillance systems: was it designed for surveillance, burden of disease or vaccine effectiveness?

- Where is this type of surveillance implemented (countries, which kind of health systems (private or public etc))?
- Investigate whether these ICD-coded surveillance systems reported syndromic data (including influenza-like-illness (ILI), severe acute respiratory infection (SARI), and acute respiratory infection (ARI)), or disease-specific for influenza, SARS-CoV-2, and RSV.
- Which ICD code or set of codes were used as proxies for syndromic data? Compare the trends from ICD-coded data (including a subset of lab-confirmed data) and trends from sentinel syndromic surveillance systems (using ILI, SARI, and ARI cases definitions).
- Assess which ICD code/sets of codes best correlated with lab-confirmed data for surveillance objectives.
- For those studies describing the use of ICD-coded data for surveillance, look for combinations of ICD-codes that are sensitive and specific for monitoring ILI, SARI, ARI, influenza, RSV, and SARS-CoV-2 trends coming from other surveillance systems.
- Assess the limitations of the use of ICD-coded data within influenza, SARS-CoV-2, and RSV surveillance systems to reach the objectives of surveillance, burden of disease and vaccine effectiveness.

Methodology:

This commissioned study proposal will follow a rapid review methodology.

Eligibility Criteria

Inclusion Criteria	Exclusion Criteria
Studies reporting on surveillance systems utilising ICD coded data	Studies reporting on surveillance systems utilising non-ICD-coded data
Studies reporting on human influenza, SARS-CoV-2, and RSV surveillance systems	Studies reporting on non-influenza, SARS-CoV-2, and RSV surveillance systems
Studies reporting on whether surveillance systems capture surveillance data, burden of disease data, or vaccine effectiveness data.	Studies reporting on aspects of surveillance systems unrelated to data capture, the burden of disease, or vaccine effectiveness (e.g., governance, funding)

Data Sources

During December 2022, different databases (* still to be confirmed but may include EMBASE, medRxiv, and MEDLINE®, COVID-END and Web of Science) will be searched to identify studies relevant to this study that are in English. Studies that are both published and unpublished will be included in the retrieval to ensure publication bias is avoided. Peer-reviewed published articles will be reviewed first, and if necessary, grey literature may be included at a later stage.

Search Strategy

Search terms used in the search strategy will be decided during December 2022. The strategy will include both key and alternative terms that relate to the research topic, and these will be decided upon during preliminary scoping literature searches.

Alongside this, a senior librarian based at the University of Edinburgh – who has experience in systematic review search strategies - will be included in the development of a search strategy. The results of this search will be documented in the Appendix of the rapid review.

Study Records:

Data Management

Study articles identified during the search will be stored and managed in COVIDENCE, which is an online software used for the management of articles in systematic reviews. Any duplicate records will be discarded using COVIDENCE.

Selection Process

Following the common systematic review methodology, two independent reviews will undertake title and abstract screening of articles identified in the literature search. Two independent reviewers will also undertake a full-text screening, to assess whether the identified articles meet this review's eligibility criteria. Finally, a PRISMA diagram will be included in the rapid review to depict the article selection process.

Data Collection Process

Articles that meet this review's eligibility criteria will have data collected by two independent reviewers. The extracted data will be stored in a standard extraction spreadsheet.

Data Items

Key characteristics of each included article will be extracted, including study location, and the relevant objectives in the context of this review.

Data Synthesis

A narrative synthesis will be undertaken to highlight the findings of this review.

Reference List

Boender, T.S., Cai, W., Schranz, M., Kocher, T., Wagner, B., Ullrich, A., Buda, S., Zöllner, R., Greiner, F., Diercke, M. and Grabenhenrich, L. (2022). Using routine emergency department data for syndromic surveillance of acute respiratory illness, Germany, week 10 2017 until week 10 2021. *Eurosurveillance*, *27*(27). P.2100865.

Buda, S., Tolksdorf, K., Schuler, E., Kuhlen, R. and Haas, W. (2017). Establishing an ICD-10 code based SARI-surveillance in Germany–description of the system and first results from five recent influenza seasons. *BMC public health*, *17*(612). DOI 10.1186/s12889-017-4515-1

ICD. (2022). International Statistical Classification of Diseases and Related Health Problems (ICD). *World Health Organisation.* [online] Available at: https://www.who.int/standards/classifications/classification-of-diseases

Appendix S2: Search strategies

Ovid MEDLINE(R) and In-Process, In-Data-Review & Other Non-Indexed Citations <1946 to January 10, 2023>

1 exp "International Classification of Diseases"/ 9271

2 (International Classification of Diseases or ICD?9 or ICD?10 or ICD?11 or acute respiratory illness code*).af. 24053

3 exp Respiratory Syncytial Viruses/ or exp Respiratory Syncytial Virus Infections/ 13246

4 (Respiratory Syncytial Vir* or RSV or ((Respiratory Syncytial Vir* or RSV) adj2 infection*)).tw,kf. 20542

5 exp SARS-CoV-2/ or exp COVID-19/ 206266

6 (SARS-CoV-2 or covid?19).tw,kf. 103180

7 exp Influenza, Human/ 56570

8 exp Influenza A Virus, H1N1 Subtype/ or exp Influenza Vaccines/ 39015

9 (influenza* or influenza?like?illness* or ILI).tw,kf. 132495

10 exp Respiratory Tract Infections/ 583413

11 (severe acute respiratory infection* or SARI or acute respiratory infection* or ARI).tw,kf. 8958

12 exp Public Health Surveillance/ or exp Population Surveillance/ or exp Sentinel Surveillance/ 74263

13 (surveillance or monitor* or survey* or incidence* or prevalence* or infection rate or morbidity or mortality or cause of death or frequenc* or burden* or occurrence* or policy or hospitali*).tw,kf. 5641751

14 1 or 2 24053

15 or/3-11 708642

16 12 or 13 5661354

17 14 and 15 and 16 696

Global Health <1973 to 2023 Week 01>

1 ("International Classification of Diseases" or ICD-9 or ICD-10 or ICD-11 or acute respiratory illness code*).ti,ab. 6976

2 exp human respiratory syncytial virus/ 6815

3 (Respiratory Syncytial Vir* or RSV or ((Respiratory Syncytial Vir* or RSV) adj2 infection*)).ti,ab. 8587

4 exp severe acute respiratory syndrome coronavirus 2/ 96388

5 exp coronavirus disease 2019/ 93079

6 (SARS-CoV-2 or covid?19).ti,ab. 40059

7 exp influenza/ 37867

8 exp Influenza A virus subtype H1N1/ or exp influenza a/ 21041

9 (influenza* or influenza?like?illness* or ILI).ti,ab. 51283

10 respiratory diseases/ 158073

11 (severe acute respiratory infection* or SARI or acute respiratory infection* or ARI).ti,ab. 5001

12 exp surveillance/ or sentinel surveillance/ or syndromic surveillance/ 56510

13 (surveillance or monitor* or survey* or incidence* or prevalence* or infection rate or morbidity or mortality or cause of death or frequenc* or burden* or occurrence* or policy or hospital* or sentinel*).ti,ab. 1422656

14 or/2-11 287798

15 12 or 13 1434371

16 1 and 14 and 15 1124

Embase <1980 to 2023 Week 01>

1 exp "international classification of diseases"/ or exp icd-10/ or exp icd-11/ or exp icd-9/ 76911

2 respiratory tract infection/ or exp respiratory tract disease/ or exp viral respiratory tract infection/ 2776638

3 exp human respiratory syncytial virus/ or exp human respiratory syncytial virus a/ 7685

4 exp Human respiratory syncytial virus/ or exp respiratory syncytial virus infection/ 13011

5 exp severe acute respiratory syndrome/ or exp severe acute respiratory syndrome coronavirus 2/ 89178

6 exp coronavirus disease 2019/ 285116

7 exp influenza/ or exp influenza a/ or exp "influenza a (h1n1)"/ or exp "influenza a (h2n2)"/ 100732

8 exp monitoring/ 996213

9 exp disease surveillance/ or exp health survey/ or exp public health surveillance/ 297165

10 or/2-7 3005233

11 8 or 9 996213

12 1 and 10 and 11 609

Table S1. Characteristics of 77 studies included in this review.

Study	Country	Study aim(s) / objective(s)	Study Design types	Study period (seasons reviewed)	Population under study
Abdel-Hady (2018)	Oman	To estimate the incidence of influenza-associated hospitalizations and in-hospital death in Oman.	Cross-sectional study	1 January 2012 to 31 December 2015	Patients attending one of the 11 provincial public hospitals of Oman
Alchikh (2019)	Germany	To assess the diagnostic accuracy of ICD codes and lab reports (standard surveillance) compared with a prospective quality management programme at a Berlin children's hospital	Observational case study	December 2009 to April 2015	All infants and children presenting to the ED at the Charité Department of Pediatrics, Berlin children's hospital
Amodio (2014)	Italy	To assess the relationship between the ICD codes for influenza used in hospital discharge records collected in Sicily and influenza circulation as observed by several European virological surveillance systems, using a large population-based database	Cross-sectional study	2007, 2008 and 2011	Patients hospitalized in Sicily
Antoon (2024)	USA	To determine the accuracy of ICD-10 influenza discharge diagnosis codes in the pediatric ED and inpatient settings	Cohort study	1 December 2016 to 31 March 2020	Children under 18 years presenting to the ED or inpatient settings with fever and/or respiratory symptoms at 7 US pediatric medical centers affiliated with the Centers for Disease Control

Study	Country	Study aim(s) / objective(s)	Study Design types	Study period (seasons reviewed)	Population under study
					and Prevention–sponsored New Vaccine Surveillance Network
Aysert-Yildiz (2019)	Turkey	To identify the types and subtypes of influenza leading to ILI, and to determine the contribution of other respiratory viruses to ILI using the ICD-10 code-based and lab-confirmed ILI surveillance in the ER	Cross-sectional study	1 December 2013 to 1 May 2015	Patients aged >18 years who were hospitalized for at least 24 hours in the Emergency Room of Gazi University Hospital and met the European Center for Disease Prevention and Control (ECDC) ILI criteria
Azziz-Baumgartner (2012)	Argentina	To estimate the excess influenza-associated pneumonia and influenza (P&I) and respiratory and circulatory illness (R&C) deaths during 2002–2009 and excess hospitalizations during 2005–2008	Observational case study	2002 to 2009 (May to October)	Hospitalized persons and persons who died with diagnosis of pneumonia and influenza respiratory and circulatory illness
Bagarella (2022)	Italy	To implement the EWMA chart and compare the performance of two predictor families (i.e., routinely collected structured and unstructured ED data, based on the ICD-9-CM codes and free text description of symptoms, respectively) for early identification of the appearance of the first cases of SARS-CoV-2	Observational case study	1 January 2011 to 9 December 2021	Patients with respiratory symptoms on ED visits in Lombardy

Study	Country	Study aim(s) / objective(s)	Study Design types	Study period (seasons reviewed)	Population under study
		infection, as well as the subsequent epidemic waves, that had occurred in an area of Lombardy (Melegnano, Italy)			
Bellazzini (2011)	USA	To demonstrate the use of ED syndromic surveillance in the setting of a novel and unexpected H1N1 influenza outbreak	Cross-sectional study	1 September 2008 to 27 May 2009	Patients visiting urban academic ED
Bernadou (2023)	France	To estimate the burden of influenza on the French hospital system by examining the proportion of severe acute respiratory infections (SARI) attributable to influenza	Observational case study	1 July 2012 to 30 June 2018	All persons in France having been discharged from hospital during the study period
Betancourt (2007)	USA	To determine whether the data in a military syndromic surveillance system accurately reflects patient visits in the population that represents active-duty military, retirees, and their family members in the Washington, DC metropolitan area.	Cross-sectional study	September 1999 to August 2000	Active and retired military and their families in the Washington DC area
Bhatt (2023)	USA	To examine the utility of Google Trends search activity on RSV to predict changes in RSV-related hospitalizations in children in the United States in 2019	Cross-sectional study	1 January 2019 to 31 December 2019	All paediatric hospitalizations (aged <21 years)

Study	Country	Study aim(s) / objective(s)	Study Design types	Study period (seasons reviewed)	Population under study
Blatt (2024)	USA	To describe the geographic progression of RSV bronchiolitis among infants across four health care systems located in different US regions, both pre– and post–COVID-19 pandemic	Cross-sectional study	4 October 2015 to 25 June 2023	All infants having accessed healthcare service from 3 health care systems: State University of New York Health System in Syracuse, NY; Duke University Health System in Durham, NC; Renown Children's Hospital in Reno, NV; and Tampa General Hospital/University of South Florida in Tampa, FL.
Boender (2022)	Germany	To explore the potential of routine emergency department data for syndromic surveillance of acute respiratory illness in Germany.	Observational case study	6 March 2017 to 13 March 2021	Routine patient attendance data from emergency departments
Bouckaert (2023)	Belgium	To document the organizational burden of RSV in a quantitative way using population-wide routinely collected Belgian hospital data (years 2017–2018)	Observational case study	2017 to 2018	RSV-associated pediatric inpatients in all hospital sites with pediatric services
Bouzille (2018)	France	To present a feasibility study on the production of accurate near-real-time estimates of ILI activity based on the clinical data warehouse, eHOP.	Observational case study	1 September 2010 to 31 August 2015 (five winter seasons beginning on	ILI-related patients at Rennes academic hospital

Study	Country	Study aim(s) / objective(s)	Study Design types	Study period (seasons reviewed)	Population under study
				the first day of September of every year and ending on 31 August of the following year)	
Bruzda (2021)	USA, Dominican Republic, El Salvador, Honduras	To analyze how the ICD-10 codes input by medical students at the Edward Via College of Osteopathic Medicine into the CREDO patient encounter logging system could act as a new syndromic surveillance tool	Cross-sectional study	November 2019 to March 2020	Appalachian and Delta regions of the US as well as Dominican Republic, Honduras and El Salvador. Population includes those who attended an Edward Via College of Osteopathic Medicine -associated hospital or clinic in those areas and were treated by a medical student.
Buda (2017)	Germany	To describe the establishment of an ICD-10-based inpatient syndromic sentinel system and its application to the analysis of five influenza seasons	Observational case study	2012 to 2016	Discharged patients and patients with respiratory illness including ICD-10 codes of primary and secondary diagnoses
Cai (2020a)	Germany	To evaluate the use of RSV-specific ICD-10 diagnosis codes for RSV surveillance	Observational case study	2007 to 2017 (week 40/2007 to week 13/2017)	Patients hospitalization with any of the three RSV-specific ICD-10 code diagnoses as primary discharge diagnosis

Study	Country	Study aim(s) / objective(s)	Study Design types	Study period (seasons reviewed)	Population under study
Cai (2020b)	Germany	To identify risk factors, in particular underlying medical conditions as risk factors, for hospitalized RSV disease and its severe outcomes based on the ICD-10-codebased surveillance data	Cohort study	2009 to 2018 (week 01/2009 to week 20/2018)	SARI and RSV cases from the hospital network of ICOSARI
Chow (2020)	USA	To examine the respiratory and non-respiratory diagnoses reported for adults hospitalized with laboratory-confirmed influenza between 2010 and 2018 in the US	Cross-sectional study	1 October to 30 April of the 2010-11 and 2017-18 influenza seasons.	Adult residents of the FluSurv-NET catchment area hospitalized with lab-confirmed influenza
Cocchio (2023)	Italy	To analyze the temporal trends and characteristics of hospitalization related to RSV in the Veneto region (Italy) in the period between 2007 and 2021	Cross-sectional study	1 January 2007 to 31 December 2021	All hospital admissions among the population of Veneto residents
Cocoros (2023)	USA	To assess the electronic medical record–based surveillance algorithm for COVID-19–like illness (CLI) performance in 5 Massachusetts medical practice groups compared with statewide counts of confirmed cases	Cross-sectional study	February 2020 to November 2022	CLI cases and patients with clinical encounters from 5 multisite clinical practice groups in eastern Massachusetts
Eick-Cost (2022)	USA	To evaluate the performance metrics of 3 COVID-19 case definitions and COVID-like	Cross-sectional study	1 March 2020 to 28 February 2021	Department of Defense health care beneficiaries

Study	Country	Study aim(s) / objective(s)	Study Design types	Study period (seasons reviewed)	Population under study
		illnesses for ambulatory encounters among Department of Defense (DOD) beneficiaries			
Elkin (2016)	USA	To determine the accuracy of ICD-9 diagnoses using laboratory confirmed cases as the gold standard	Cohort study	October 2000 to March 2006	Patients with an ICD-9-CM diagnosis of influenza and patients with laboratory confirmed Influenza from Mayo Clinic
Farah (2023)	Lebanon	To estimate the number and rate of influenza-associated respiratory hospitalization in Lebanon during five influenza seasons (2015–2016 to 2019–2020) by age and province of residence in addition to estimate the influenza burden by level of severity.	Cross-sectional study	2015 to 2020	SARI cases from sentinel surveillance system and the Ministry of Public Health (MoPH) hospital billing database.
Feemster (2016)	USA	To develop and validate a surveillance definition for healthcare-associated influenza-like illness (HA-ILI) based upon electronic health record (EHR) data	Cohort study	October to April 2012–2013 through 2014–2015	Children (<6 years) presenting to any of 31 clinics within a pediatric primary care network
Fung (2023)	USA	To estimate the incidence of long COVID and long Flu among Medicare patients using the WHO consensus definition	Cohort study	1 April 2020 to 30 June 2021	Medicare beneficiaries

Study	Country	Study aim(s) / objective(s)	Study Design types	Study period (seasons reviewed)	Population under study
Gerbier-Colomban (2014)	France	To evaluate whether intra-hospital surveillance systems including an automatic syndromic surveillance system can detect the onset of influenza epidemics earlier than regional influenza surveillance networks	Observational case study	1 June 2007 to 31 March 2011	Patients older than 15 years of age who visited the adult ED of the North Hospital Group, Lyon University Hospitals
Giordani (2024)	Italy	To define a specific combination of codes to identify the COVID-19 hospitalizations within the Hospital Information System and to investigate the risk factors associated with mortality due to COVID-19 among patients admitted to Italian hospitals in 2020	Cohort study	1 January 2020 to 31 December 2020	Patients hospitalized due to COVID-19
Girit (2017)	Turkey	To study hospital-based surveillance and risk factors of influenza-A in children hospitalized with ILI symptoms over two influenza seasons	Cross-sectional study	December 2013 to May 2014; and December 2014 to May 2015	All children hospitalized with a diagnosis of ILI
Gundlapalli (2021)	USA	To assess documentation of diagnoses co-occurring with an ICD-10 code for COVID-19 (U07.1) on US death certificates	Cross-sectional study	1 January 2020 to 22 February 2021	Entire US population: deaths that were attributed to COVID-19 on death certificates
Habbous (2023)	Canada	To examine year-over-year trends in hospitalizations and emergency	Cross-sectional study	1 January 2017 to 20 May 2022	Hospital admissions and ED visits for influenza and RSV

Study	Country	Study aim(s) / objective(s)	Study Design types	Study period (seasons reviewed)	Population under study
		department (ED) visits associated with various respiratory viruses in Ontario, Canada			
Hagiwara (2022)	Japan	To describe the characteristics of laboratory-confirmed and clinically diagnosed influenza patients aged ≥60 years who presented to outpatient and inpatient care settings, and explore associated complications, clinical outcomes and related health resource utilization during hospital stay.	Cross-sectional study	6 September 2010 to 1 September 2019	Outpatients and inpatients
Ishiguro (2024)	Japan	To develop and validate claims-based algorithms for identifying hospitalized patients with coronavirus disease (COVID-19) and the disease severity	Cross-sectional study	1 January 2020 to 31 December 2021	All patients at the National Center for Global Health and Medicine Hospital
Johnson (2022)	USA	To describe testing behaviors, symptoms, impact, vaccination status, and case ascertainment during the COVID-19 pandemic using integrated data sources.	Cross-sectional study	June 2020 to May 2021	All UCHealth patients
Keck (2014)	USA	To describe the characteristics and utility of this new EHR-based surveillance system and present an evaluation of its ILI surveillance component	Cross-sectional study	1 May 2009 to 29 March 2010	Eligible people from the American Indian (AI) and Alaska Native (AN) population; 60% of the AI/AN population are

Study	Country	Study aim(s) / objective(s)	Study Design types	Study period (seasons reviewed)	Population under study
					eligible, and this is 1.5m people
Khanh (2021)	Vietnam	To estimate the national disease burden of influenza-associated hospitalization	Cross-sectional study	2014 to 2016	SARI and ARI cases hospitalized in public hospitals in Vietnam
Leiner (2023)	Germany	To conduct an extended analysis of SARI and COVID-19 cases covering four pandemic waves to report on patient characteristics and respective outcomes and compare pre-pandemic with pandemic time periods, utilizing a large-scale administrative dataset derived from the Initiative of Quality Medicine	Cohort study	Pre-pandemic period: 1 January 2019 to 3 March 2020; and Pandemic period: 4 March 2020 to 31 December 2021	COVID-19 and SARI inpatients from 421 German acute care hospitals
Light (2008)	USA	To determine if there is an association between seasonal virology data and the incidence of ICD-9 coded hospitalizations for RSV lower respiratory tract illness	Cross-sectional study	2001 to 2004	All Children (patients) <24 months of age with either a principal or secondary discharge diagnosis related to RSV
Loubet (2024)	France	To describe the occurrence and outcomes of RSV-related hospital stays in adults in France through a nation-wide database analysis	Observational case study	1 September 2012 to 31 August 2021	Adults (≥18 years) hospitalized with an RSV-related condition
Mad Tahir (2023)	Malaysia	To estimate the economic burden of influenza on older adults (those aged ≥60 years) in Malaysia from the provider's perspective.	Cross-sectional study	2010 to 2020	Older adult influenza patients in Malaysia

Study	Country	Study aim(s) / objective(s)	Study Design types	Study period (seasons reviewed)	Population under study
Marsden-Haug (2007)	USA	To determine whether ICD-9 groupings used to detect ILI within an automated syndromic system correlate with respiratory virus laboratory test results in the same population	Cross-sectional study	October 2000 to December 2004	Patients from all permanent military treatment facilities
Matias (2017)	USA	To estimate seasonal influenza- and RSV-attributable hospitalizations in the US from 1997 to 2009 according to age and risk status	Observational case study	1 October 1997 to 31 March 2009	All patients of any age who were recorded in the US Nationwide Inpatient Sample (NIS)
Mattiuzzi (2023)	USA	To obtain current statistics on direct mortality caused by Influenza viruses in the US	Cross-sectional study	2018 to 2020	US residents in the CDC Wonder Online database
McLeod (2009)	New Zealand	To investigate whether the Wellington ED Respiratory Syndromic Surveillance System might have provided an early warning of the influenza outbreak in Wellington schools during 2005, and as a result provided the opportunity for an earlier or more effective public health response.	Cross-sectional study	1 January 2004 to 31 December 2006	Patients discharged with any event of respiratory syndrome from Wellington Hospital ED
McMurry (2024)	USA	To validate and test an artificial intelligence (AI)–based natural language processing (NLP)	Cohort study	1 March 2020 to 31 May 2022	Pediatric patients who presented to a pediatric ED at

Study	Country	Study aim(s) / objective(s)	Study Design types	Study period (seasons reviewed)	Population under study
		pipeline for detecting COVID-19 symptoms from physician notes in pediatric patients			a large academic children's hospital
Milliren (2023)	USA	To examine trends in coronavirus disease 2019 (COVID-19) hospitalizations and severity of disease among children using administrative data from pediatric hospitals in the United States	Cross-sectional study	5 April 2020 to 27 August 2022	Pediatric patients with COVID-19 admitted in pediatric hospitals
Mira-Iglesias (2022)	Spain	To better quantify the incidence of RSV-associated hospitalized disease by season, age, and birth month	Observational case study	2014 to 2018	Hospitalized infant patients resident in the catchment area of one of the participating public hospitals
Moore (2011)	Australia	To identify which emergency department disease codes best correlated with confirmed influenza cases and to determine if these same codes would be useful in the non-influenza season	Observational case study	6 July 2001 to 7 August 2009	Individuals presenting to Emergency Departments (ED) of two major hospitals in Melbourne and lab-confirmed influenza cases for the corresponding period from NIDS.
Motlogeloa (2023)	South Africa	To explore the timing of onset and cessation of the acute infectious respiratory disease season and the relevant thresholds in South Africa	Cross-sectional study	2008 to 2019	All persons hospitalized at Baragwanath hospital, and all persons having made a medical insurance claim through Discovery Medical Insurance Scheme

Study	Country	Study aim(s) / objective(s)	Study Design types	Study period (seasons reviewed)	Population under study
Moura (2024)	Canada	To assess the operating characteristics of ICD-10 code U07.1 using the Canadian COVID-19 Emergency Department Rapid Response Network (CCEDRRN) registry linked to administrative diagnostic codes	Cross-sectional study	1 March 2020 to August 2021	Individuals from eight Canadian provinces (British Columbia, Alberta, Manitoba, Saskatchewan, Quebec, Ontario, Nova Scotia, and New Brunswick) who presented to 51 urban and rural emergency departments
Murray (2023)	Australia	To investigate the impact of the introduction of rapid testing on hospital administrative coding for influenza	Observational case study	1 January 2016 to 30 June 2019	Adult inpatients at The Royal Melbourne Hospital
Ortiz (2014)	USA	To assess the extent to which restricting influenza testing to adults hospitalized with pneumonia could underestimate the total burden of hospitalized influenza disease	Observational case study	January 2003 to March 2009	Adult population in datasets
Pattie (2009)	USA	To evaluate the sensitivity, specificity, and positive predictive value of body temperature measurements ≥100.5◦F in relationship with laboratory confirmation of influenza and other ILI pathogens	Cross-sectional study	1 December 2007 to 29 February 2008	ILI cases recorded in AHLTA, which supports more than 9.1 million TRICARE beneficiaries worldwide

Study	Country	Study aim(s) / objective(s)	Study Design types	Study period (seasons reviewed)	Population under study
Pumarola (2023)	Spain	To estimate the clinical and economic burden of severe influenza in Spain from season 2008/09 to season 2017/18 across distinct age groups	Observational case study	January 2008 to December 2018	Patients hospitalized with a diagnosis of influenza in Spanish Public Hospitals
Ramos-Rincon (2024)	Spain	To analyze five influenza seasons in Spain, from 2016–2017 to 2020–2021, aiming to (a) describe the clinical characteristics of hospitalizations with a diagnosis of influenza, (b) assess the associated morbidity (hospitalization rates and ICU admissions rate), mortality and cost of influenza hospitalizations in different age groups and (c) analyze the risk factors for mortality	Cross-sectional study	1 July 2016 to 30 June 2021	Patient with hospitalizations related to influenza
Ramsay (2010)	Australia	To develop a real-time process, using syndromic (monitoring of selected International Classification of Disease (ICD-10) codes to the Emergency Department (ED)) and laboratory data sources (requests for respiratory polymerase chain reaction (PCR) tests), to monitor ILI presentations and admissions.	Cross-sectional study	April to September, from 2006 until 2008	ILI and influenza presentations and admissions through the health service at a 440-bed tertiary referral hospital in Melbourne, Victoria, Australia

Study	Country	Study aim(s) / objective(s)	Study Design types	Study period (seasons reviewed)	Population under study
Reed (2014)	USA	To describe and compare influenza-associated complications among adults hospitalized with H1N1pdm09 and seasonal influenza	Case series	2005 to 2010. Seasonal influenza (hospitalized 1 October 2005 to 14 April 2009); or H1N1pdm09 (hospitalized 15 April 2009 to 30 April 2010)	Adult patients hospitalized with laboratory-confirmed influenza.
Ricoca Peixoto (2023)	Portugal	To identify factors associated with COVID-19 intensive care unit (ICU) admission and death among hospitalized cases in Portugal, and variations from the first to the second wave in Portugal, March–December 2020	Cohort study	Period 1: 2 March to 30 September 2020; Period 2: 1 October to 16 December 2020	All hospitalized cases of laboratory-confirmed COVID-19
Saleh (2024)	USA	To determine the prevalence and odds ratio of erythema multiforme (EM) after COVID-19 infection or vaccination	Cross-sectional study	January 2020 to December 2022	All patients admitted to University of Florida Health and Shands Hospital
San-Roman-Montero (2019)	Spain	To assess differences of the burden of severe or complicated influenza illness and inpatient hospital fatality in each influenza season	Cross-sectional study	1 October 2009 to 30 September 2015	All hospital discharges with diagnosis of influenza

Study	Country	Study aim(s) / objective(s)	Study Design types	Study period (seasons reviewed)	Population under study
		from 2009 to 2015 through the hospital discharge database.			
Schirmer (2010)	USA	To demonstrate the performance of VA ESSENCE for detection of 2008–09 seasonal and pandemic H1N1 2009 influenza in VA healthcare facilities	Cross-sectional study	28 September 2008 to 25 April 2009 (CDC 2008–2009 flu season weeks 40–16); 26 April 2009 to 31 July 2009 (CDC 2008–2009 flu season weeks 17–30)	US veteran patients
Shappell (2023)	USA	To examine the impact of commonly used case definitions for coronavirus disease 2019 (COVID-19) hospitalizations on case counts and outcomes	Cohort study	1 March 2020 to 1 March 2022	All adults hospitalized at 5 Massachusetts acute-care hospitals
Sigmundsdottir (2010)	Iceland	To study the changes in surveillance of influenza in Iceland and data collected during the 2009 influenza A(H1N1) pandemic	Cross-sectional study	1 April to December 2009	All patients with suspected ILI or diagnosed confirmed influenza
Sivakumaran (2023)	Wales	To characterise microbiology testing and results associated with emergency admissions for acute exacerbation of COPD (AECOPD), and determine the accuracy of	Cross-sectional study	1 December 2016 to 30 November 2018	All NHS hospital admitted patients in Wales

Study	Country	Study aim(s) / objective(s)	Study Design types	Study period (seasons reviewed)	Population under study
		ICD-10 codes in retrospectively identifying laboratory-confirmed respiratory pathogens in this setting			
Sotomayor (2018)	Chile	To assess the burden of influenza-associated deaths and hospitalizations between 2012-2014 using SARI surveillance	Cross-sectional study	1 January 2012 to 31 December 2014	Persons hospitalized or deceased (obtained from records of national hospitalizations and deaths) due to influenza or pneumonia
Taylor (2016)	UK	To use time-series regression methods to analyze the available data and estimate the RSV-attributable medical burden among children in the UK in terms of GP episodes, hospitalizations, and deaths due to respiratory diseases and other outcomes, by age, during the period 1995-2009	Observational case study	1 July 1995 to 2009	Young children admitted to hospital with respiratory disease
Thiam (2022)	France	To describe the epidemiological characteristics of COVID-19-related visits to both emergency departments (EDs) and the network of emergency general practitioners known as SOS Me´decins (SOSMed) in France from 17 February to 28 June 2020	Cross-sectional study	17 February to 28 June 2020	COVID-19-related visits to both emergency departments (EDs) and the network of emergency general practitioners known as SOS Médecins (SOSMed)

Study	Country	Study aim(s) / objective(s)	Study Design types	Study period (seasons reviewed)	Population under study
Torres (2023)	Portugal	To describe the establishment of the Portuguese SARI sentinel surveillance system and its application in the 2021/2022 season (week 40 2021 to week 20 2022) and compare the evolution of SARI cases with the COVID-19 and influenza activity in two regions of Portugal.	Cross-sectional study	28 September 2021 to 22 May 2022 (week 40 2021 (2021/40) and week 20 2022 (2022/20)	SARI cases reported in two Portuguese university general hospitals, Centro Hospitalar Universitario de São João and Centro Hospitalar Universitario Lisboa Central
Trucchi (2019)	Italy	To assess the health and economic burden of influenza and its complications in terms of annual incidence rates of ILIs and LRTIs requiring ED access, whether followed by hospitalization or not, and expenditures, according to age and comorbidities.	Observational case study	1 November 2011 to 31 October 2017	Ligurian population, Genoa, Italy
Wan Puteh (2023)	Malaysia	To estimate the cost of influenza management, using the Casemix system	Cross-sectional study	2016, 2017 and 2018	SARI cases reported in two sources: an urban teaching hospital Hospital Cancellor Tuanku Muhriz (HCTM) and Ministry of Health (MoH) inpatient hospitals database.
Wan Puteh (2024)	Malaysia	To determine the trends and costs of influenza in Malaysia	Cross-sectional study	2016, 2017 and 2018	SARI and ILI cases reported in: the Virology Unit of the Institute of Medical Research

Study	Country	Study aim(s) / objective(s)	Study Design types	Study period (seasons reviewed)	Population under study
					(IMR); the National Public Health Laboratory (NPHL); the University of Malaya Medical Center (UMMC); Kumpulan Perubatan Johor (KPJ) Hospitals (Damansara and Ampang Puteri); and Hospital Canselor Tuanku Muhriz Universiti Kebangsaan Malaysia (HCTM UKM)
Wander (2023)	USA	To determine the rates, clinical setting, risk factors, and symptoms associated with the documentation of International Statistical Classification of Diseases Tenth Revision (ICD-10), code U09.9 for post–COVID-19 condition after acute infection	Cohort study	1 October 2021 to 31 January 2023	388,980 US veterans with positive test results for SARS-CoV-2 during the Omicron era
Wells (2022)	Scotland	To investigate how patient diagnosis codes, recorded within electronic healthcare data sets, could be used to capture SARI trends in Scotland	Observational case study	2017 to 2022	All inpatient hospital admission (SARI cases) in Scotland
Whittaker (2023)	Norway	To critically appraise and further develop the surveillance of people hospitalized with COVID-19, by	Cross-sectional study	17 February 2020 to 1 May 2022	Hospitalized Covid-19 patients

Study	Country	Study aim(s) / objective(s)	Study Design types	Study period (seasons reviewed)	Population under study
		comparing data on hospitalized COVID-19 patients from two national health registries in Norway from February 2020 to May 2022			
Wick (2023)	Germany	To quantify the number of RSV-related hospitalizations in children ≤2 years of age and to determine corresponding resource use and costs in Germany	Cross-sectional study	2019 to 2022	RSV-associated hospitalization cases in children
Widgren (2010)	Denmark	To describe the findings from the Danish influenza surveillance system set up to monitor influenza-associated hospitalizations during the 2009/2010 pandemic season	Cross-sectional study	January 2004 to 2010	All in-patient hospitalizations with the selected ICD-10 codes as primary or secondary diagnosis

Table S2a. Quality Assessment of 65 cross-sectional/observational studies included in this review

Author (Year)	1. Was the sample frame appropriate to address the target population?	2. Were study participants recruited in an appropriate way?	3. Was the sample size adequate?	4. Were the study subjects and setting described in detail?	5. Was data analysis conducted with sufficient coverage of the identified sample?	6. Were valid methods used for the identification of the condition?	7. Was the condition measured in a standard, reliable way for all participants?	8. Was there appropriate statistical analysis?	9. Was the response rate adequate, and if not, was the low response rate managed appropriately?	Overall appraisal
Abdel-Hady (2018)	Yes	Yes	Yes	Yes	Yes	Yes	Yes	Yes	NA	High
Alchikh (2019)	Yes	Yes	Yes	Yes	Yes	Yes	Yes	Yes	NA	High
Amodio (2014)	Yes	Yes	Yes	Yes	Yes	Yes	Yes	No	NA	Moderate
Aysert-YIldIz (2019)	Yes	Yes	Yes	Yes	Yes	Yes	Yes	No	NA	Moderate
Azziz-Baumgartner (2013)	Yes	Yes	Yes	Yes	Yes	Yes	Yes	Yes	NA	High
Bagarella (2022)	Yes	Yes	Yes	Yes	Yes	Yes	Yes	No	NA	Moderate

Author (Year)	1. Was the sample frame appropriate to address the target population?	2. Were study participants recruited in an appropriate way?	3. Was the sample size adequate?	4. Were the study subjects and setting described in detail?	5. Was data analysis conducted with sufficient coverage of the identified sample?	6. Were valid methods used for the identification of the condition?	7. Was the condition measured in a standard, reliable way for all participants?	8. Was there appropriate statistical analysis?	9. Was the response rate adequate, and if not, was the low response rate managed appropriately?	Overall appraisal
Bellazzini (2011)	Yes	Yes	Yes	Yes	Yes	Yes	Yes	No	NA	Moderate
Bernadou (2023)	Yes	Yes	Yes	Yes	Yes	Yes	Yes	Yes	NA	High
Betancourt (2007)	Yes	Yes	Yes	Yes	Yes	Yes	Yes	No	NA	Moderate
Bhatt (2023)	Yes	Yes	Yes	Yes	Yes	Yes	Yes	Yes	NA	High
Blatt (2024)	Yes	Yes	Yes	No	Yes	Yes	Yes	Yes	NA	Moderate
Boender (2022)	Yes	Yes	Yes	Yes	Yes	Yes	Yes	Yes	NA	High
Bouckaert (2023)	Yes	Yes	Yes	Yes	Yes	Yes	Yes	No	NA	Moderate
Bouzille (2018)	Yes	Yes	Yes	Yes	Yes	Yes	Yes	Yes	NA	High

Author (Year)	1. Was the sample frame appropriate to address the target population?	2. Were study participants recruited in an appropriate way?	3. Was the sample size adequate?	4. Were the study subjects and setting described in detail?	5. Was data analysis conducted with sufficient coverage of the identified sample?	6. Were valid methods used for the identification of the condition?	7. Was the condition measured in a standard, reliable way for all participants?	8. Was there appropriate statistical analysis?	9. Was the response rate adequate, and if not, was the low response rate managed appropriately?	Overall appraisal
Bruzda (2021)	Yes	Yes	Yes	Yes	Yes	Yes	Yes	No	NA	Moderate
Buda (2017)	Yes	Yes	Yes	Yes	Yes	Yes	Yes	No	No	Moderate
Cai (2020a)	Yes	Yes	Yes	Yes	Yes	Yes	Yes	Yes	NA	High
Chow (2020)	Yes	Yes	Yes	Yes	Yes	Yes	Yes	No	NA	Moderate
Cocchio (2023)	Yes	Yes	Yes	Yes	Yes	Yes	Yes	Yes	NA	High
Cocoros (2023)	Yes	Yes	Yes	Yes	Yes	Yes	Yes	No	NA	Moderate
Eick-Cost (2022)	Yes	Yes	Yes	Yes	Yes	Yes	Yes	No	NA	Moderate
Farah (2023)	Yes	Yes	Yes	Yes	Yes	Yes	Yes	Yes	NA	High

**Author (Year)**	**1. Was the sample frame appropriate to address the target population?**	**2. Were study participants recruited in an appropriate way?**	**3. Was the sample size adequate?**	**4. Were the study subjects and setting described in detail?**	**5. Was data analysis conducted with sufficient coverage of the identified sample?**	**6. Were valid methods used for the identification of the condition?**	**7. Was the condition measured in a standard, reliable way for all participants?**	**8. Was there appropriate statistical analysis?**	**9. Was the response rate adequate, and if not, was the low response rate managed appropriately?**	**Overall appraisal**
Gerbier-Colomban (2014)	Yes	Yes	Yes	Yes	Yes	Yes	Yes	No	NA	Moderate
Girit (2017)	Yes	Yes	Yes	Yes	Yes	Yes	Yes	Yes	NA	High
Gundlapalli (2021)	Yes	Yes	Yes	Yes	Yes	Yes	Yes	No	NA	Moderate
Habbous (2023)	Yes	Yes	Yes	Yes	Yes	Yes	Yes	Yes	NA	High
Hagiwara (2022)	Yes	Yes	Yes	Yes	Yes	Yes	Yes	Yes	NA	High
Ishiguro (2024)	Yes	Yes	Yes	Yes	Yes	Yes	Yes	Yes	NA	High
Johnson (2022)	Yes	Yes	Yes	Yes	Yes	Yes	Yes	Yes	NA	High
Keck (2014)	Yes	Yes	Yes	Yes	Yes	Yes	Yes	No	NA	Moderate

Author (Year)	1. Was the sample frame appropriate to address the target population?	2. Were study participants recruited in an appropriate way?	3. Was the sample size adequate?	4. Were the study subjects and setting described in detail?	5. Was data analysis conducted with sufficient coverage of the identified sample?	6. Were valid methods used for the identification of the condition?	7. Was the condition measured in a standard, reliable way for all participants?	8. Was there appropriate statistical analysis?	9. Was the response rate adequate, and if not, was the low response rate managed appropriately?	Overall appraisal
Khanh (2021)	Yes	Yes	Yes	Yes	Yes	Yes	Yes	Yes	NA	High
Light (2008)	Yes	Yes	Yes	Yes	Yes	Unclear	Unclear	No	NA	Moderate
Loubet (2024)	Yes	Yes	Yes	Yes	Yes	Yes	Yes	No	NA	Moderate
Mad Tahir (2023)	Yes	Yes	Yes	Yes	Yes	Yes	Yes	Yes	NA	High
Marsden-Haug (2007)	Yes	Yes	Yes	Yes	Yes	Yes	Yes	No	NA	Moderate
Matias (2017)	Yes	Yes	Yes	Yes	Yes	Yes	Yes	Yes	NA	High
Mattiuzzi (2023)	Yes	Yes	Yes	Yes	Yes	Yes	Yes	No	NA	Moderate

Author (Year)	1. Was the sample frame appropriate to address the target population?	2. Were study participants recruited in an appropriate way?	3. Was the sample size adequate?	4. Were the study subjects and setting described in detail?	5. Was data analysis conducted with sufficient coverage of the identified sample?	6. Were valid methods used for the identification of the condition?	7. Was the condition measured in a standard, reliable way for all participants?	8. Was there appropriate statistical analysis?	9. Was the response rate adequate, and if not, was the low response rate managed appropriately?	Overall appraisal
McLeod (2009)	Yes	Yes	Yes	Yes	Yes	Yes	Yes	No	NA	Moderate
Milliren (2023)	Yes	Yes	Yes	Yes	Yes	Yes	Yes	Yes	NA	High
Mira-Iglesias (2022)	Yes	Yes	Yes	Yes	Yes	Yes	Yes	Yes	NA	High
Moore (2011)	Yes	Yes	Yes	Yes	Yes	Yes	Yes	No	NA	Moderate
Motlogeloa (2023)	Yes	Yes	Yes	Yes	Yes	Yes	Yes	Yes	NA	High
Moura (2024)	Yes	Yes	Yes	Yes	Yes	Yes	Yes	Yes	NA	High
Murray (2023)	Yes	Yes	Yes	Yes	Yes	Yes	Yes	Yes	NA	High

Author (Year)	1. Was the sample frame appropriate to address the target population?	2. Were study participants recruited in an appropriate way?	3. Was the sample size adequate?	4. Were the study subjects and setting described in detail?	5. Was data analysis conducted with sufficient coverage of the identified sample?	6. Were valid methods used for the identification of the condition?	7. Was the condition measured in a standard, reliable way for all participants?	8. Was there appropriate statistical analysis?	9. Was the response rate adequate, and if not, was the low response rate managed appropriately?	Overall appraisal
Ortiz (2014)	Yes	Yes	Yes	Yes	Yes	Yes	Yes	Yes	NA	High
Pattie (2009)	Yes	Yes	Yes	Yes	Yes	Yes	Yes	No	NA	Moderate
Pumarola (2023)	Yes	Yes	Yes	Yes	Yes	Yes	Yes	Yes	NA	High
Ramos-Rincon (2024)	Yes	Yes	Yes	Yes	Yes	Yes	Yes	Yes	NA	High
Ramsay (2010)	Yes	Yes	Yes	Yes	Yes	Yes	Yes	No	Unclear	Moderate
Saleh (2024)	Yes	Yes	Yes	No	Yes	Yes	Unclear	Yes	NA	Moderate
San-Roman-Montero (2019)	Yes	Yes	Yes	Yes	Yes	Yes	Yes	Yes	NA	High

Author (Year)	1. Was the sample frame appropriate to address the target population?	2. Were study participants recruited in an appropriate way?	3. Was the sample size adequate?	4. Were the study subjects and setting described in detail?	5. Was data analysis conducted with sufficient coverage of the identified sample?	6. Were valid methods used for the identification of the condition?	7. Was the condition measured in a standard, reliable way for all participants?	8. Was there appropriate statistical analysis?	9. Was the response rate adequate, and if not, was the low response rate managed appropriately?	Overall appraisal
Schirmer (2010)	Yes	Yes	Yes	Yes	Yes	Yes	Yes	Yes	NA	High
Sigmundsdottir (2010)	Yes	Yes	Yes	Yes	Yes	Yes	Yes	No	NA	Moderate
Sivakumaran (2023)	Yes	Yes	Yes	Yes	Yes	Yes	Yes	Yes	NA	High
Sotomayor (2018)	Yes	Yes	Yes	Yes	Yes	Yes	Yes	Yes	NA	High
Taylor (2016)	Yes	Yes	Yes	Yes	Yes	Yes	Yes	Unclear	NA	Moderate
Thiam (2022)	Yes	Yes	Yes	Yes	Yes	Yes	Yes	No	NA	Moderate
Torres (2023)	Yes	Yes	Yes	Yes	Yes	Yes	Yes	No	NA	High
Trucchi (2019)	Yes	Yes	Yes	Yes	Yes	Yes	Yes	No	NA	High

Author (Year)	1. Was the sample frame appropriate to address the target population?	2. Were study participants recruited in an appropriate way?	3. Was the sample size adequate?	4. Were the study subjects and setting described in detail?	5. Was data analysis conducted with sufficient coverage of the identified sample?	6. Were valid methods used for the identification of the condition?	7. Was the condition measured in a standard, reliable way for all participants?	8. Was there appropriate statistical analysis?	9. Was the response rate adequate, and if not, was the low response rate managed appropriately?	Overall appraisal
Wan Puteh (2023)	Yes	Yes	Yes	Yes	Yes	Yes	Yes	No	NA	Moderate
Wan Puteh (2024)	Yes	Yes	Yes	Yes	Yes	Yes	Yes	No	NA	Moderate
Wells (2022)	Yes	Yes	Yes	Yes	Yes	Yes	Yes	Yes	NA	High
Whittaker (2023)	Yes	Yes	Yes	Yes	Yes	Yes	Yes	No	NA	Moderate
Wick (2023)	Yes	Yes	Yes	Yes	Yes	Yes	Yes	No	NA	Moderate
Widgren (2010)	Yes	Yes	Yes	Yes	Yes	Yes	Yes	Yes	NA	High

Table S2b. Quality Assessment of 11 cohort studies included in this review.

Author (Year)	1. Were the two groups similar and recruited from the same population?	2. Were the exposures measured similarly to assign people to both exposed and unexposed groups?	3. Was the exposure measured in a valid and reliable way?	4. Were confounding factors identified?	5. Were strategies to deal with confounding factors stated?	6. Were the groups/ participants free of the outcome at the start of the study (or at the moment of exposure)?	7. Were the outcomes measured in a valid and reliable way?	8. Was the follow up time reported and sufficient to be long enough for outcomes to occur?	9. Was follow up complete, and if not, were the reasons to loss to follow up described and explored?	10. Were strategies to address incomplete follow up utilized?	11. Was appropriate statistical analysis used?	Overall appraisal
Antoon (2024)	Yes	Yes	Yes	Yes	Yes	Yes	Yes	Yes	Yes	NA	Yes	High
Cai (2020b)	Yes	Yes	Yes	Yes	Yes	Yes	Yes	Yes	Unclear	Unclear	Yes	High
Elkin (2016)	Yes	Yes	Yes	No	No	Yes	Yes	Unclear	Unclear	Unclear	Unclear	Low
Feemster (2016)	Yes	Yes	Yes	No	No	Yes	Yes	Yes	Unclear	Yes	Yes	Moderate
Fung (2023)	Yes	Yes	Yes	Yes	Yes	Yes	Yes	Yes	Yes	NA	Yes	High
Giordani (2024)	Yes	Yes	Yes	Yes	Yes	Yes	Unclear	Yes	Unclear	Yes	Yes	High

Author (Year)	1. Were the two groups similar and recruited from the same population?	2. Were the exposures measured similarly to assign people to both exposed and unexposed groups?	3. Was the exposure measured in a valid and reliable way?	4. Were confounding factors identified?	5. Were strategies to deal with confounding factors stated?	6. Were the groups/ participants free of the outcome at the start of the study (or at the moment of exposure)?	7. Were the outcomes measured in a valid and reliable way?	8. Was the follow up time reported and sufficient to be long enough for outcomes to occur?	9. Was follow up complete, and if not, were the reasons to loss to follow up described and explored?	10. Were strategies to address incomplete follow up utilized?	11. Was appropriate statistical analysis used?	Overall appraisal
Leiner (2023)	Yes	Yes	Yes	Yes	Yes	Yes	Yes	Yes	Unclear	Yes	Yes	High
McMurry (2024)	Yes	Yes	Yes	Unclear	Yes	Yes	Yes	Yes	Unclear	No	Yes	Moderate
Ricoca Peixoto (2023)	Yes	Yes	Yes	Yes	Yes	Yes	Yes	Yes	Yes	NA	Yes	High
Shappell (2023)	Yes	Yes	Yes	Yes	Unclear	Yes	Yes	Yes	Unclear	Unclear	Yes	Moderate
Wander (2023)	Yes	Yes	Yes	Yes	Yes	Yes	Yes	Yes	Yes	Unclear	Yes	High

Table S2c. Quality Assessment of one case series study included in this review.

Author (Year)	1. Were there clear criteria for inclusion in the case series?	2. Was the condition measured in a standard, reliable way for all participants included in the case series?	3. Were valid methods used for identification of the condition for all participants included in the case series?	4. Did the case series have consecutive inclusion of participants?	5. Did the case series have complete inclusion of participants?	6. Was there clear reporting of the demographics of the participants in the study?	7. Was there clear reporting of clinical information of the participants?	8. Were the outcomes or follow-up results of cases clearly reported?	9. Was there clear reporting of the presenting site(s) / clinic(s) demographic information?	10. Was statistical analysis appropriate?	Overall appraisal
Reed (2014)	Yes	Yes	Yes	Yes	Unclear	Yes	Yes	Yes	Yes	Yes	High

Table S3. Characteristics of ICD-coded surveillance systems and objectives

**Author (year)**	**Location**	**Name of surveillance system**	**Setting of surveillance system**	**ICD codes used**	**Syndrome(s) under study (ILI/ SARI/ ARI)**	**ICD-Code(s) used (ILI/ SARI/ ARI)**	**Specific disease under study (Influenza/ RSV/ COVID-19)**	**ICD-Code(s) used (Influenza/ RSV/ COVID-19)**
Systems Recording Surveillance Data								
Antoon (2024) [1]	USA	NVSN	Public and Private	ICD-10	NA	NA	Influenza	J09, J09X, J09X1, J09X2, J09X3, J09X9, J10, J100, J1001, J1008, J101, J102, J108, J1081, J1082, J1083, J1089, J11, J110, J1100, J1108, J111, J112, J118, J1181, J1182, J1183, J1189
Bagarella (2022) [2]	Italy	Structured and unstructured data from visits	Public	ICD-9-CM	Respiratory syndrome (including	00322, 0203, 0204, 0205, 0212, 0221,	N/A	N/A

Author (year)	Location	Name of surveillance system	Setting of surveillance system	ICD codes used	Syndrome(s) under study (ILI/ SARI/ ARI)	ICD-Code(s) used (ILI/ SARI/ ARI)	Specific disease under study (Influenza/ RSV/ COVID-19)	ICD-Code(s) used (Influenza/ RSV/ COVID-19)
		to 3 EDs of a local health assessment unit in Lombardy			SARS-CoV-2 cases)	0340, 0391, 0521, 0551, 05679, 09889, 1048, 1124, 1140, 1144, 1145, 11505, 1173, 1304, 1363, 38100, 38101, 38102, 38103, 38104, 38105, 38106, 3814, 38150, 38151, 38200, 38201, 38202, 3821, 3822, 3823, 3824, 3829, 460, 4610, 4611, 4612, 4613, 4618, 4619, 462, 463,		

Author (year)	Location	Name of surveillance system	Setting of surveillance system	ICD codes used	Syndrome(s) under study (ILI/ SARI/ ARI)	ICD-Code(s) used (ILI/ SARI/ ARI)	Specific disease under study (Influenza/ RSV/ COVID-19)	ICD-Code(s) used (Influenza/ RSV/ COVID-19)
						46400, 46401, 46410, 46411, 46420, 46421, 46430, 46431, 4644, 46450, 46451, 4650, 46500, 4658, 4659, 4660, 46611, 46619, 470, 4710, 4711, 4718, 4719, 4720, 4721, 4722, 4730, 4731, 4732, 4733, 4738, 4739, 47400, 47401, 47402, 47410, 47411, 47412, 4748, 4749, 475, 4760,		

Author (year)	Location	Name of surveillance system	Setting of surveillance system	ICD codes used	Syndrome(s) under study (ILI/ SARI/ ARI)	ICD-Code(s) used (ILI/ SARI/ ARI)	Specific disease under study (Influenza/ RSV/ COVID-19)	ICD-Code(s) used (Influenza/ RSV/ COVID-19)
						4761, 4770, 4771, 4772, 4778, 4779, 4780, 47811, 47819, 47821, 47822, 47824, 47829, 47830, 47831, 47834, 4784, 4785, 4786, 47874, 47875, 47879, 4788, 4789, 480, 4800, 4801, 4802, 4803, 4808, 4809, 481, 4820, 4821, 4822, 48232, 48239, 48240, 48241, 48242, 48249, 48282,		

Author (year)	Location	Name of surveillance system	Setting of surveillance system	ICD codes used	Syndrome(s) under study (ILI/ SARI/ ARI)	ICD-Code(s) used (ILI/ SARI/ ARI)	Specific disease under study (Influenza/ RSV/ COVID-19)	ICD-Code(s) used (Influenza/ RSV/ COVID-19)
						48283, 48289, 4829, 4830, 4831, 4838, 4845, 4847, 4848, 485, 486, 4870, 4871, 4878, 48881, 48882, 48889, 490, 4910, 4911, 49120, 49122, 4918, 4919, 4928, 49300, 49301, 49302, 49310, 49311, 49312, 49322, 49381, 49382, 49390, 49391, 49392, 4940, 4941, 4950, 4951, 4952,		

Author (year)	Location	Name of surveillance system	Setting of surveillance system	ICD codes used	Syndrome(s) under study (ILI/ SARI/ ARI)	ICD-Code(s) used (ILI/ SARI/ ARI)	Specific disease under study (Influenza/ RSV/ COVID-19)	ICD-Code(s) used (Influenza/ RSV/ COVID-19)
						4953, 4954, 4955, 4956, 4957, 4958, 4959, 500, 501, 502, 503, 504, 505, 5060, 5061, 5062, 5063, 5064, 5069, 5070, 5071, 5078, 5080, 5081, 5082, 5088, 5089, 5100, 5109, 5110, 5111, 51181, 51189, 5119, 5120, 5121, 5122, 51281, 51282, 51283, 51284, 51289, 5130,		

Author (year)	Location	Name of surveillance system	Setting of surveillance system	ICD codes used	Syndrome(s) under study (ILI/ SARI/ ARI)	ICD-Code(s) used (ILI/ SARI/ ARI)	Specific disease under study (Influenza/ RSV/ COVID-19)	ICD-Code(s) used (Influenza/ RSV/ COVID-19)
						5131, 514, 515, 5160, 516+O991, 5162, 5163, 51630, 51631, 51632, 51633, 51634, 51635, 51636, 51637, 5164, 5165, 51661, 51662, 51663, 51664, 51669, 5168, 5169, 5171, 5178, 5180, 5181, 5182, 5183, 5184, 51851, 51852, 51853, 5187, 51881, 51882, 51883, 51884, 51889, 51900,		

**Author (year)**	**Location**	**Name of surveillance system**	**Setting of surveillance system**	**ICD codes used**	**Syndrome(s) under study (ILI/ SARI/ ARI)**	**ICD-Code(s) used (ILI/ SARI/ ARI)**	**Specific disease under study (Influenza/ RSV/ COVID-19)**	**ICD-Code(s) used (Influenza/ RSV/ COVID-19)**
						51901, 51909, 51911, 51919, 5192, 5193, 5194, 5198, 5199, 53084, 769, 78600, 78609, 78630, 78631, 78639, 78650, 78651, 78652, 78659, 7867, 7869, 7991, 99731, 99739, 99811, 9982, V4614, 48041		
Bellazzini (2011) [3]	USA	ED syndromic surveillance system	Public	ICD-9	ILI	487.00, 487.10, 487.80, 79.99, 79.89, 780.60, 490.00,	N/A	N/A

**Author (year)**	**Location**	**Name of surveillance system**	**Setting of surveillance system**	**ICD codes used**	**Syndrome(s) under study (ILI/ SARI/ ARI)**	**ICD-Code(s) used (ILI/ SARI/ ARI)**	**Specific disease under study (Influenza/ RSV/ COVID-19)**	**ICD-Code(s) used (Influenza/ RSV/ COVID-19)**
						486.00, 465.80, 465.90, 466.00, 786.20, 460.00, 485.00, 461.90, 466.19		
Betancourt (2007) [4]	USA	ESSENCE	Public	ICD-9	ILI / SARI / ARI	465.9, 462, 079.99, 382.9, 461.9, 466.0, 786.2, 486. 460, 490, 034.0, 382.00, 463, 461.0, 786.05, 487.1, 381.01, 461.8, 465.8, 464.4, 780.6, 034.1,	Influenza	487.1

**Author (year)**	**Location**	**Name of surveillance system**	**Setting of surveillance system**	**ICD codes used**	**Syndrome(s) under study (ILI/ SARI/ ARI)**	**ICD-Code(s) used (ILI/ SARI/ ARI)**	**Specific disease under study (Influenza/ RSV/ COVID-19)**	**ICD-Code(s) used (Influenza/ RSV/ COVID-19)**
						780.31, 038.11, 038.0, 038.40, 038.19, 038.8, 002.0, 038.10, 038.43, 038.49, 038.42, 038.41, 038.2, 002.1, 003.1, 038.3, 038.44, 002.9, 558.9, 787.91, 787.01, 008.8, 787.03, 535.50, 787.02, 535.00, 009.0, 009.1, 555.9, 008.69, 787.3, 787.99, 536.2,		

Author (year)	Location	Name of surveillance system	Setting of surveillance system	ICD codes used	Syndrome(s) under study (ILI/ SARI/ ARI)	ICD-Code(s) used (ILI/ SARI/ ARI)	Specific disease under study (Influenza/ RSV/ COVID-19)	ICD-Code(s) used (Influenza/ RSV/ COVID-19)
						009.2, 535.40, 009.3, 578.0, 005.9		
Bhatt (2023) [5]	USA	2019 HCUP-KID	Public	ICD-10	N/A	N/A	RSV	J12.1, B97.4, J20.5, and J21.0
Blatt (2024) [6]	USA	EHR data from four health care systems: SUNY Health System; Duke University Health System; Renown Children's Hospital; and Tampa General Hospital/	Public and Private	ICD-10	N/A	N/A	RSV	B97.4, J12.1, J20.5, J21.0, J21.8 or J21.9

**Author (year)**	**Location**	**Name of surveillance system**	**Setting of surveillance system**	**ICD codes used**	**Syndrome(s) under study (ILI/ SARI/ ARI)**	**ICD-Code(s) used (ILI/ SARI/ ARI)**	**Specific disease under study (Influenza/ RSV/ COVID-19)**	**ICD-Code(s) used (Influenza/ RSV/ COVID-19)**
		University of South Florida						
Boender (2022) [7]	Germany	The AKTIN ED Data Registry and the ESEG project	Public	ICD-10	ILI / SARI / ARI	ARI: J00-J22, J44.0, B34.9, U07.1, U07.2<br>SARI: J09-J22, U07.1, U07.2<br>ILI Probable Case: J06.-, J12.8, J12.9, J18.-, J22, R50.8, R50.9<br>ILI Confirmed Case: J09, J10.-, J11	RSV / COVID-19	RSV Probable Case: J12.8, J12.9, J18.-, J20.8, J20.9, J21.8, J21.9, J22<br>RSV Confirmed Case: J12.1, J20.5, J21.0, B97.4<br>COVID-19 Probable Case: U07.1, U07.2<br>COVID-19 Confirmed Case: U07.1

Author (year)	Location	Name of surveillance system	Setting of surveillance system	ICD codes used	Syndrome(s) under study (ILI/ SARI/ ARI)	ICD-Code(s) used (ILI/ SARI/ ARI)	Specific disease under study (Influenza/ RSV/ COVID-19)	ICD-Code(s) used (Influenza/ RSV/ COVID-19)
Bouzille (2018) [8]	France	eHOP clinical data warehouse technology of the academic hospital of Rennes	Public	ICD-10	ILI	J09.x, J10.x or J11.x	Influenza	J09.xx and J10.xx
Buda (2017) [9]	Germany	Hospitals belonging to the HELIOS Kliniken Gmb	Private	ICD-10	SARI	J09 – J22	Influenza / RSV	Influenza: J09.xx and J10.xx<br>RSV: J12.1.
Cai (2020a) [10]	Germany	SEEDARE; IC OSARI; and from the virological surveillance at the Robert Koch Institute	Public and Private	ICD-10	ARI	J06 (J06, J06.0, J06.8, J06.9), J11 (J11, J11.0, J11.1, J11.8), J12 (J12, J12.8, J12.9), J18 (J18, J18.0, J18.8,	RSV	J12.1, J20.5, and J21.0

Author (year)	Location	Name of surveillance system	Setting of surveillance system	ICD codes used	Syndrome(s) under study (ILI/ SARI/ ARI)	ICD-Code(s) used (ILI/ SARI/ ARI)	Specific disease under study (Influenza/ RSV/ COVID-19)	ICD-Code(s) used (Influenza/ RSV/ COVID-19)
						J18.9), J20 (J20, J20.8, J20.9), J21 (J21, J21.8, 21.9), J22, and B34.9		
Cai (2020b) [11]	Germany	ICOSARI database	Private	ICD-10	SARI	J09-J22 (J09-J11, J12-J18, J20, J21, J22)	Influenza / RSV	Influenza: J09.xx and J10.xx<br>RSV: J12.1, J20.5, J21.0
Chow (2020) [12]	USA	The US FluSurv-NET	Public	ICD-9-CM & ICD-10	Acute respiratory or non-respiratory diagnoses	ICD-9-CM codes: 480-488, 460-519 and 390-459; and ICD-10 codes: J09-J18, J00-J99 and I00-I99	Influenza	ICD-9-CM codes: 487-488<br>ICD-10 codes: J09-J11

Author (year)	Location	Name of surveillance system	Setting of surveillance system	ICD codes used	Syndrome(s) under study (ILI/ SARI/ ARI)	ICD-Code(s) used (ILI/ SARI/ ARI)	Specific disease under study (Influenza/ RSV/ COVID-19)	ICD-Code(s) used (Influenza/ RSV/ COVID-19)
Cocchio (2023) [13]	Italy	Hospital discharge records of Veneto's database	Public and Private	ICD-9-CM	N/A	N/A	RSV	079.6; 466.11; and 480.1
Cocoros (2023) [14]	USA	ESP public health surveillance platform	Public	ICD-10-CM	N/A	N/A	COVID-19–like illness (CLI)	B34.2, B97.29, U07.1
Eick-Cost (2022) [15]	USA	DMSS	Public	ICD-10-CM	COVID-like illness (CLI)	B34.2, B97.21, B97.29, J12.81, J20.8, J22, J40, J80, R05, J00, J06.9, J12.9, J16.8, J17, J18.0, J18.1, J18.8, J18.9, J20.9,	COVID-19	U07.1

**Author (year)**	**Location**	**Name of surveillance system**	**Setting of surveillance system**	**ICD codes used**	**Syndrome(s) under study (ILI/ SARI/ ARI)**	**ICD-Code(s) used (ILI/ SARI/ ARI)**	**Specific disease under study (Influenza/ RSV/ COVID-19)**	**ICD-Code(s) used (Influenza/ RSV/ COVID-19)**
						J84.111, R06.0, R06.00, R06.02, R06.03, R06.09, R43.0, R43.2, R50.9		
Elkin (2016) [16]	USA	Biosense biosurveillance system	Private	ICD-9-CM	ILI	487 and sub-codes 487.XX	Influenza	487.1
Feemster (2016) [17]	USA	EHRs from a large paediatric network of clinics	Public and Private	ICD-9-CM	ILI	382.0, 382.9, O38.8, O38.9, 780.6	Influenza	478, 487.1, 487.8
Gerbier-Colomban (2014) [18]	France	UrgIndex surveillance system at the North Hospital	Public	ICD-10	SARI	J09, J10, J11, J10.0, J10.1, J10.8, J11.0,	Influenza	J09.xx and J10.xx

**Author (year)**	**Location**	**Name of surveillance system**	**Setting of surveillance system**	**ICD codes used**	**Syndrome(s) under study (ILI/ SARI/ ARI)**	**ICD-Code(s) used (ILI/ SARI/ ARI)**	**Specific disease under study (Influenza/ RSV/ COVID-19)**	**ICD-Code(s) used (Influenza/ RSV/ COVID-19)**
		Group of the Lyon University Hospitals				J11.1, and J11.8		
Johnson (2022) [19]	USA	Biobank at CCPM	Public	ICD-10	N/A	N/A	COVID-19	U07.1
Leiner (2023) [20]	Germany	IQM network	Public	ICD-10	SARI	J09-J22	COVID-19	U07.1
Light (2008) [21]	USA	Florida Department of Health's epidemiology surveillance network	Public	ICD-9-CM	N/A	N/A	RSV	079.6; 466.11; and 480.1
Marsden-Haug (2007) [22]	USA	An automated ILI surveillance report	Public	ICD-9	ILI	079.89, 079.99, 460, 462, 464.00, 464.10, 464.20, 465.0,	RSV	466.11, 480.1

**Author (year)**	**Location**	**Name of surveillance system**	**Setting of surveillance system**	**ICD codes used**	**Syndrome(s) under study (ILI/ SARI/ ARI)**	**ICD-Code(s) used (ILI/ SARI/ ARI)**	**Specific disease under study (Influenza/ RSV/ COVID-19)**	**ICD-Code(s) used (Influenza/ RSV/ COVID-19)**
		incorporated into ESSENCE				465.8, 465.9, 466.0, 466.11, 466.19, 478.9, 480.0, 480.1, 480.2, 480.8, 480.9, 484.8, 485, 486, 487.0, 487.1, 487.8, 490, 780.6, 784.1, 786.2		
McLeod (2009) [23]	New Zealand	Wellington ED Respiratory Syndromic Surveillance System	Public	ICD-10	Respiratory syndrome	J00-J06, J09-J18, J20-J22, J30, J31, J32, J35, J36, J37, J39, J40-47, J60-J68, J70, J80-J84, J85-J86, J90, J91, J93, J94, J96,	Influenza	J09, J10.0, J10.1, J10.8

**Author (year)**	**Location**	**Name of surveillance system**	**Setting of surveillance system**	**ICD codes used**	**Syndrome(s) under study (ILI/ SARI/ ARI)**	**ICD-Code(s) used (ILI/ SARI/ ARI)**	**Specific disease under study (Influenza/ RSV/ COVID-19)**	**ICD-Code(s) used (Influenza/ RSV/ COVID-19)**
						J98, R04, R05, R06, R07		
McMurry (2024) [24]	USA	an open-source AI–based natural language processing pipeline that includes a large language model	Public	ICD-10	N/A	N/A	COVID-19	U07.1
Moore (2011) [25]	Australia	SynSurv	Public	ICD-10	ILI	J06, J11, J22, B34, J18	Influenza	J11
Pattie (2009) [26]	USA	AHLTA	Public	ICD-9	ILI	79.99, 382.9, 460, 461.9, 465.9, 466, 486, 490, 780.6, 786.2	Influenza	487.1

**Author (year)**	**Location**	**Name of surveillance system**	**Setting of surveillance system**	**ICD codes used**	**Syndrome(s) under study (ILI/ SARI/ ARI)**	**ICD-Code(s) used (ILI/ SARI/ ARI)**	**Specific disease under study (Influenza/ RSV/ COVID-19)**	**ICD-Code(s) used (Influenza/ RSV/ COVID-19)**
Ramsay (2010) [27]	Australia	Community-based Victorian GP surveillance program	Public	ICD-10	ILI	J11.1, B34.9, J22, J06.9, J00, J42, J20.9, J18.0, R50.9	N/A	N/A
Reed (2014) [28]	USA	EIP Influenza Surveillance Network	Public	ICD-9	SARI	480–488; 460–519; 390–519	Influenza	488.11
Schirmer (2010) [29]	USA	ESSENCE	Public	ICD-9-CM	ILI	466.0, 466.11, 466.19, 490., 786.2, 478.9, 780.6, 780.60, 780.64, 780.65, 487.0, 487.1, 487.8, 480.0, 480.1, 480.2, 480.8, 480.9, 484.8, 485, 486, 784.1, 460.,	N/A	N/A

**Author (year)**	**Location**	**Name of surveillance system**	**Setting of surveillance system**	**ICD codes used**	**Syndrome(s) under study (ILI/ SARI/ ARI)**	**ICD-Code(s) used (ILI/ SARI/ ARI)**	**Specific disease under study (Influenza/ RSV/ COVID-19)**	**ICD-Code(s) used (Influenza/ RSV/ COVID-19)**
						462., 464.00, 464.10, 464.20, 465.0, 465.8, 465.9, 079.89, 079.99		
Sigmundsdottir (2010) [30]	Iceland	CHS-CDC	Public	ICD-10	ILI	J10, J10.0, J10.1, J10.8, J11, J11.0, J11.1, J11.8 and U05.9	Influenza	J10.xx
Thiam (2022) [31]	France	SurSaUD®	Public and Private	ICD-10	SARI	B34.2, B97.2, U04.9, U07.1, U07.10, U07.11, U07.12, U07.14, and U07.15	COVID-19	U07.1, U07.10, U07.12, and U07.14

Author (year)	Location	Name of surveillance system	Setting of surveillance system	ICD codes used	Syndrome(s) under study (ILI/ SARI/ ARI)	ICD-Code(s) used (ILI/ SARI/ ARI)	Specific disease under study (Influenza/ RSV/ COVID-19)	ICD-Code(s) used (Influenza/ RSV/ COVID-19)
Torres (2023) [32]	Portugal	SARI sentinel surveillance system	Public	ICD-10	SARI	R05, R06, R07.0, R13, R50.9, R51, M79.1, R53.1, R53.81, R53.83; J09-J18, J20-J22, B34.9, A49.9, J40, 41, I40.9, U07.1, U07.2	COVID-19 / Influenza	COVID-19: U07.1<br>Influenza: J09, J10
Trucchi (2019) [33]	Italy	Syndromic Surveillance System	Public	ICD-9	ILI	ICD-9-CM codes 052.1, 112.4, 136.3, 480-487	N/A	N/A
Wells (2022) [34]	Scotland	SARI surveillance system: using data from	Public	ICD-10	SARI	J09-J22, J80, U07.1, U07.2 and J04	Influenza / COVID-19	Influenza: J09, J10<br>COVID-19: U07.1

**Author (year)**	**Location**	**Name of surveillance system**	**Setting of surveillance system**	**ICD codes used**	**Syndrome(s) under study (ILI/ SARI/ ARI)**	**ICD-Code(s) used (ILI/ SARI/ ARI)**	**Specific disease under study (Influenza/ RSV/ COVID-19)**	**ICD-Code(s) used (Influenza/ RSV/ COVID-19)**
		SMR01 and RAPID						
Whittaker (2023) [35]	Norway	NPR and NoPaR	Public	ICD-10	N/A	N/A	COVID-19	U07.1
Systems Recording Burden of Disease Data								
Abdel-Hady (2018) [36]	Oman	Nabdh Al Shifa (computerized online system)	Public	ICD-10	SARI	J09-J18	Influenza	J09-J18
Azziz-Baumgartner (2013) [37]	Argentina	PAHO's mortality databases and Argentina's hospitalization databases	Public	ICD-10	Unspecified	J10-J18, I00-I99 and J00-J99	Influenza	N/A
Bouckaert (2023) [38]	Belgium	B-HDDS	Public	ICD-10	N/A	N/A	RSV	B97.4, J12.1, J20.5, and J21.0

Author (year)	Location	Name of surveillance system	Setting of surveillance system	ICD codes used	Syndrome(s) under study (ILI/ SARI/ ARI)	ICD-Code(s) used (ILI/ SARI/ ARI)	Specific disease under study (Influenza/ RSV/ COVID-19)	ICD-Code(s) used (Influenza/ RSV/ COVID-19)
Fung (2023) [39]	USA	CMS Virtual Research Data Center	Public	ICD-10	N/A	N/A	(long) COVID-19 / Influenza	COVID-19: U07.1<br>long COVID: B94.8<br>influenza: J09, J10, and J11
Habbous (2023) [40]	Canada	DAD and NACRS	Public	ICD-10	N/A	N/A	Influenza / RSV	Influenza: J09, J10.0, J10.1, J10.8<br>RSV: J12.1, J20.5, J21.0
Mad Tahir (2023) [41]	Malaysia	Casemix system (of a teaching hospital in Malaysia)	Public	ICD-10	Mild, moderate, and severe influenza	J09, J10.0, J10.1, J10.8, J11.0, J11.1, J11.8, J12.8, and J12.9	Influenza	J09, J10.0, J10.1, J10.8
Matias (2017) [42]	USA	Hospitalisation data from US	Public	ICD-9	Respiratory diseases	480-488; 460-519, 079,	Influenza / RSV	Influenza: 488

**Author (year)**	**Location**	**Name of surveillance system**	**Setting of surveillance system**	**ICD codes used**	**Syndrome(s) under study (ILI/ SARI/ ARI)**	**ICD-Code(s) used (ILI/ SARI/ ARI)**	**Specific disease under study (Influenza/ RSV/ COVID-19)**	**ICD-Code(s) used (Influenza/ RSV/ COVID-19)**
		Nationwide Inpatient Sample; and virological data from FluView				786.0-786.4, 786.7-786.9; 460-519; 390-519; 038, 771.81		RSV: 466.11, 480.1
Moura (2024) [43]	Canada	CCEDRRN registry linked to administrative diagnostic codes	Public	ICD-10	N/A	N/A	COVID-19	U07.1
Murray (2023) [44]	Australia	NATA approved, batch testing, in-house real-time PCR platform	Public	ICD-10	N/A	N/A	Influenza	J09 or J10

Author (year)	Location	Name of surveillance system	Setting of surveillance system	ICD codes used	Syndrome(s) under study (ILI/ SARI/ ARI)	ICD-Code(s) used (ILI/ SARI/ ARI)	Specific disease under study (Influenza/ RSV/ COVID-19)	ICD-Code(s) used (Influenza/ RSV/ COVID-19)
Pumarola (2023) [45]	Spain	PHDB for Spain	Public	ICD-9-MC & ICD-10-ES	ILI / SARI	ICD-9-MC: 480–488, 517.1, 460–519, 390–459, 460–519<br>ICD-10-ES: J09-J18, J00-J99, I00-I99	Influenza	ICD-9-MC: 488; ICD-10-ES: J09, J10
Ricoca Peixoto (2023) [46]	Portugal	Clinical registries of Portuguese NHS hospitals	Public	ICD-10	N/A	N/A	COVID-19	U07.1
Saleh (2024) [47]	USA	IDR i2b2 platform	Public and Private	ICD-10	N/A	N/A	COVID-19	U07.1
Sivakumaran (2023) [48]	Wales	SAIL Databank	Public	ICD-10	N/A	N/A	Influenza / RSV	Influenza: J09, J10, J11

**Author (year)**	**Location**	**Name of surveillance system**	**Setting of surveillance system**	**ICD codes used**	**Syndrome(s) under study (ILI/ SARI/ ARI)**	**ICD-Code(s) used (ILI/ SARI/ ARI)**	**Specific disease under study (Influenza/ RSV/ COVID-19)**	**ICD-Code(s) used (Influenza/ RSV/ COVID-19)**
								RSV: J12.1, J20.5, J21.0, B97.4
Taylor (2016) [49]	UK	CPRD; HES database; ONS database; UK national surveillance system at PHE	Public	ICD-10	Respiratory outcomes (unspecified)	J00-09, J09-18, J20-22, J40, J41-47, J00-99, I00-99	RSV	J12.1
Wan Puteh (2023) [50]	Malaysia	Casemix system	Public	ICD-10	ILI / SARI	B97.8, H66.90, J00, J01.9, J06.9, J09.X1, J09.X2, J09.X3, J10.0, J11.0, J18.0, J20.9, J40, R05, R50.9	Influenza	J09.X1, J09.X2, J09.X3, J10.0

**Author (year)**	**Location**	**Name of surveillance system**	**Setting of surveillance system**	**ICD codes used**	**Syndrome(s) under study (ILI/ SARI/ ARI)**	**ICD-Code(s) used (ILI/ SARI/ ARI)**	**Specific disease under study (Influenza/ RSV/ COVID-19)**	**ICD-Code(s) used (Influenza/ RSV/ COVID-19)**
Wan Puteh (2024) [51]	Malaysia	Casemix system	Public	ICD-10	ILI / SARI	B97.8, H66.90, J01.9, J06.9, J09.X1, J09.X2, J09.X3, J09.X9, J10.0, J11.0, J12.9, J18.0, J20.9, J40, R05, R50.9	Influenza	J09.X1, J09.X2, J09.X3, J10.0
Wander (2023) [52]	USA	US Department of VA's Corporate Data Warehouse and CSDR	Public	ICD-10	N/A	N/A	Post-COVID-19 condition	U09.9
Systems Recording Both Surveillance and Burden of Disease Data								
Alchikh (2019) [53]	Germany	Quality management	Public	ICD-10	ILI / SARI / ARI	J09, J10.0, J10.1, J10.8,	Influenza	J09, J10.0, J10.1, J10.8

Author (year)	Location	Name of surveillance system	Setting of surveillance system	ICD codes used	Syndrome(s) under study (ILI/ SARI/ ARI)	ICD-Code(s) used (ILI/ SARI/ ARI)	Specific disease under study (Influenza/ RSV/ COVID-19)	ICD-Code(s) used (Influenza/ RSV/ COVID-19)
		programme at a Berlin children's hospital with the Robert Koch Institute				J11.0, J11.1, J11.8, J12.0, J12.1, J12.3, J20.5, J20.6, J21.0, J21.1		
Amodio (2014) [54]	Italy	The Regional Hospital Discharge Database; WHO FluNet database	Public and Private	ICD-9-CM	ILI	480.0-480.3, 480.8, 480.9, 487.0, 487.1, 487.8	N/A	N/A
Aysert-Yildiz (2019) [55]	Turkey	GIHSN	Public and Private	ICD-10	ILI	R06, J11, J18	Influenza	J11, R06, J18
Bernadou (2023) [56]	France	PMSI	Public and Private	ICD-10	SARI	J12-J20	Influenza	J09-J11
Bruzda (2021) [57]	USA, Dominica	CREDO system	Public	ICD-10	ILI / SARI	J00, J01, J06, J09-12, J18,	NA	NA

**Author (year)**	**Location**	**Name of surveillance system**	**Setting of surveillance system**	**ICD codes used**	**Syndrome(s) under study (ILI/ SARI/ ARI)**	**ICD-Code(s) used (ILI/ SARI/ ARI)**	**Specific disease under study (Influenza/ RSV/ COVID-19)**	**ICD-Code(s) used (Influenza/ RSV/ COVID-19)**
	n Republic, El Salvador, Honduras					J20, J22, J40, R05		
Farah (2023) [58]	Lebanon	SARI sentinel surveillance system; and the Ministry of Public Health's hospital billing database	Public	ICD-10	SARI	J09-J18	Influenza	J09.xx and J10.xx
Giordani (2024) [59]	Italy	Hospital discharge records (collected by Italian MoH), linked with	Public and Private	ICD-9-CM	NA	NA	COVID-19	043, 480.4, 518.9, 519.7,

Author (year)	Location	Name of surveillance system	Setting of surveillance system	ICD codes used	Syndrome(s) under study (ILI/ SARI/ ARI)	ICD-Code(s) used (ILI/ SARI/ ARI)	Specific disease under study (Influenza/ RSV/ COVID-19)	ICD-Code(s) used (Influenza/ RSV/ COVID-19)
		National Tax Registry						
Girit (2017) [60]	Turkey	GIHSN	Public and Private	ICD-10	ILI	J00-J06, J20-J22, H66.90, J09-J18, R06.0, R06, R06.9, R06.3, R06.00, R06.09, R06.83, R06.02, R06.82, R06.2, R06.89, M79.1, R50, R50.9, R05	Influenza	J09.xx and J10.xx
Gundlapalli (2021) [61]	USA	Death certificates from CDC	Public	ICD-10	NA	NA	COVID-19	U07.1

Author (year)	Location	Name of surveillance system	Setting of surveillance system	ICD codes used	Syndrome(s) under study (ILI/ SARI/ ARI)	ICD-Code(s) used (ILI/ SARI/ ARI)	Specific disease under study (Influenza/ RSV/ COVID-19)	ICD-Code(s) used (Influenza/ RSV/ COVID-19)
Hagiwara (2022) [62]	Japan	RWD database	Public	ICD-10	N/A	N/A	Influenza	J09, J10
Ishiguro (2024) [63]	Japan	Claims data from the National Center for Global Health and Medicine Hospital	Public	ICD-10	NA	N/A	COVID-19	U07.1, B34.2
Keck (2014) [64]	USA	The Indian Health Service's Influenza Awareness System	Public	ICD-9	ILI	786.2, 780.60; 079.99; 486, 465.9, 382.9; 462; 466.19; 490; 460; 466.0; 382.00; 461.8; 461.9; 463; 464.00; 464.10; 464.20; 465.0; 465.8; 478.9;	Influenza	487.1, 487.0, 487.8

**Author (year)**	**Location**	**Name of surveillance system**	**Setting of surveillance system**	**ICD codes used**	**Syndrome(s) under study (ILI/ SARI/ ARI)**	**ICD-Code(s) used (ILI/ SARI/ ARI)**	**Specific disease under study (Influenza/ RSV/ COVID-19)**	**ICD-Code(s) used (Influenza/ RSV/ COVID-19)**
						480.9; 485; 780.6		
Khanh (2021) [65]	Vietnam	Vietnam's SARI sentinel surveillance system; Vietnam's MoH EMRs system	Public	ICD-10	SARI / ARI	J06, J10, J11, J12, J13, J14, J15, J16, J17, J18, J20, J21, J22	Influenza	J10
Loubet (2024) [66]	France	PMSI	Public and Private	ICD-10	N/A	N/A	RSV	J20.5, J21.0, J12.1, B97.4
Mattiuzzi (2023) [67]	USA	CDC WONDER Online Database	Public	ICD-10	N/A	N/A	Influenza	J09, J10.0, J10.1, J10.8
Milliren (2023) [68]	USA	Paediatric Hospital Information System	Public and Private	ICD-10	N/A	N/A	COVID-19	U07.1, J12.82

Author (year)	Location	Name of surveillance system	Setting of surveillance system	ICD codes used	Syndrome(s) under study (ILI/ SARI/ ARI)	ICD-Code(s) used (ILI/ SARI/ ARI)	Specific disease under study (Influenza/ RSV/ COVID-19)	ICD-Code(s) used (Influenza/ RSV/ COVID-19)
Mira-Iglesias (2022) [69]	Spain	VAHNSI	Public	ICD-10	ARI	J00-J06, J09-J18, J20-J22, H66.90	Influenza / RSV	influenza: J09.xx and J10.xx<br>RSV: J12.1
Motlogeloa (2023) [70]	South Africa	Data from the South African Medical Research Council Respiratory and Meningeal Pathogens Research Unit database; and the Discover Medical Insurance Scheme claims database	Public and Private	ICD-10	ARI	J00, J11.0, J11.1, J11.8	N/A	N/A

Author (year)	Location	Name of surveillance system	Setting of surveillance system	ICD codes used	Syndrome(s) under study (ILI/ SARI/ ARI)	ICD-Code(s) used (ILI/ SARI/ ARI)	Specific disease under study (Influenza/ RSV/ COVID-19)	ICD-Code(s) used (Influenza/ RSV/ COVID-19)
Ortiz (2014) [71]	USA	State Inpatient Databases from Arizona, California, and Washington; and Regional influenza surveillance data from CDC	Public	ICD-9-CM	SARI	96.7, 480–486, 518.5, 518.81, 518.82, 995.92, 785.52	Influenza	487.xx
Ramos-Rincon (2024) [72]	Spain	SNSSHD, specifically the Hospital Care Activity Record - Minimum Basic Data Set (Registro de actividades especializadas-Conjunto	Public and Private	ICD-10	SARI	J10.0	Influenza	J09, J10

Author (year)	Location	Name of surveillance system	Setting of surveillance system	ICD codes used	Syndrome(s) under study (ILI/ SARI/ ARI)	ICD-Code(s) used (ILI/ SARI/ ARI)	Specific disease under study (Influenza/ RSV/ COVID-19)	ICD-Code(s) used (Influenza/ RSV/ COVID-19)
		Mínimo Básico de datos)						
San-Roman-Montero (2019) [73]	Spain	National Surveillance System for Hospital Data (Conjunto Mínimo Básico de Datos, CMBD)	Public	ICD-9-CM	N/A	N/A	Influenza	488.1
Shappell (2023) [74]	USA	Mass General Brigham healthcare System	Public	ICD-10	N/A	N/A	COVID-19	U07.1, J12.82
Sotomayor (2018) [75]	Chile	SARI sentinel surveillance database; Records of hospital	Public and Private	ICD-10	SARI	J09-J18	Influenza	Influenza: J09.xx and J10.xx; RSV: J12.1

Author (year)	Location	Name of surveillance system	Setting of surveillance system	ICD codes used	Syndrome(s) under study (ILI/ SARI/ ARI)	ICD-Code(s) used (ILI/ SARI/ ARI)	Specific disease under study (Influenza/ RSV/ COVID-19)	ICD-Code(s) used (Influenza/ RSV/ COVID-19)
		discharges and national deaths, managed by the DSHI of the MoH of Chile						
Wick (2023) [76]	Germany	German Institute for the Hospital Remuneration System	Public	ICD-10	SARI	J09–J11, J12–J18, J20, J21, J22	RSV	J12.1, J20.5, J21.0
Widgren (2010) [77]	Denmark	Danish National Patient Registry	Public	ICD-10	SARI	G051F, G051O, H671B, J09, J091, J091A, J091B, J099, J10, J100, J101, J101A, J101B,	Influenza	J09.xx and J10.xx

Author (year)	Location	Name of surveillance system	Setting of surveillance system	ICD codes used	Syndrome(s) under study (ILI/ SARI/ ARI)	ICD-Code(s) used (ILI/ SARI/ ARI)	Specific disease under study (Influenza/ RSV/ COVID-19)	ICD-Code(s) used (Influenza/ RSV/ COVID-19)
						J101C, J108, J108A, J108B, J108C, J11, J110, J111, J111A, J111B, J111C, J118, J118A, J118B, J118C, I411A; J12, J120, J121, J122, J128, J129, J18, J180, J181, J182, J188, J189; J13, J139, J139A, J139B, J14, J149, J149A, J149B, J15, J150, J151,		

Author (year)	Location	Name of surveillance system	Setting of surveillance system	ICD codes used	Syndrome(s) under study (ILI/ SARI/ ARI)	ICD-Code(s) used (ILI/ SARI/ ARI)	Specific disease under study (Influenza/ RSV/ COVID-19)	ICD-Code(s) used (Influenza/ RSV/ COVID-19)
						J152, J153, J154, J155, J156, J156A, J157, J158, J159, J16, J160, J168, J170, J170A, J170B, J170C, J170D, J170E, J170F, J170H, J171, J171A, J171B, J171C, J171D, J172, J172A, J172B, J172C, J172D, J173, J173A,		

Author (year)	Location	Name of surveillance system	Setting of surveillance system	ICD codes used	Syndrome(s) under study (ILI/ SARI/ ARI)	ICD-Code(s) used (ILI/ SARI/ ARI)	Specific disease under study (Influenza/ RSV/ COVID-19)	ICD-Code(s) used (Influenza/ RSV/ COVID-19)
						J173B, J173C, J178, J178A, J178B, J178C, R560, J96, J960, J969		

AI – Artificial Intelligence; AHLTA – Armed Forces Health Longitudinal Technological Application; ARI – Acute Respiratory Infection; B-HDDS – Belgian Hospital Discharge Data Set; CCEDRRN – Canadian COVID-19 Emergency Department Rapid Response Network; CCPM – Colorado Center for Personalized Medicine; CDC – Center for Disease Control and Prevention; CHS-CDC – Centre for Health Security and Communicable Disease Control; CMS – Centers for Medicare and Medicaid Services; CPRD – Clinical Practice Research Datalink; CREDO – Clinical Rotation Evaluation and Documentation Organizer; CSDR – COVID-19 Shared Data Resource; DAD – Discharge Abstract Database; DMSS – Defense Medical Surveillance System; DSHI – Department of Statistics and Health Information; ED – Emergency Department; EHR – Electronic Health Record; EIP – Emerging Infections Program; ESEG – Erkennung und Steuerung Epidemischer Gefahrenlagen; ESP – Electronic medical record Support for Public health; ESSENCE – Electronic Surveillance System for the Early Notification of Community-based Epidemics; FluSurv-NET – Influenza Hospitalisation Surveillance Network; GIHSN – Global Influenza Hospital Surveillance Network; GP – General Practitioner; HCUP-KID – Healthcare Cost and Utilization Project's Kids Inpatient Database; HES – Hospital Episode Statistics; ICOSARI – ICD-10 code-based surveillance system for severe acute respiratory infections; IDR – Integrated Data Repository; ILI – Influenza-like illness; IQM – Initiative of Quality Medicine; MoH – Ministry of Health; MY-DRG – Malaysian Diagnosis Related Group; NACRS – National Ambulatory Care Reporting System; NATA – National Association of Testing Authorities; NHS – National Health Services;

NoPaR – Norwegian Pandemic Registry; NPR – Norwegian Patient Registry; NVSN – New Vaccine Surveillance Network; N/A – not available; ONS – Office of National Statistics; PAHO – Pan American Health Organization; PCR – polymerase chain reaction; PHDB – Projected Hospitalizations Database; PHE – Public Health England; PMSI – Programme de M´edicalisation des Systemes d'information; RAPID – Rapid Preliminary Inpatient Dataset; RWD – Real-World Data; SAIL – Secure Anonymized Information Linkage; SARI – Severe Acute Respiratory Infections; SEEDARE – ICD-10-based influenza and other ARI surveillance systems sentinel electronic data collection system; SMR01 – The General Acute Inpatient and Day Case; SNSSHD – Spanish National Surveillance System for Hospital Data; SUNY – State University of New York; SurSaUD – Surveillance Sanitaire des Urgences et des Décès; SynSurv – Victorian Syndromic Surveillance; VA – Veterans Affairs; VAHNSI – Valencia Hospital Network for the Study of Influenza; WHO – World Health Organization.

Table S4. Case definitions for ILI/ SARI/ ARI/ Influenza/RSV/COVID-19, and Limitations of using ICD-coded data within surveillance systems

Study	Country	Name of system	Case definition for ILI/ SARI/ ARI/ Influenza/RSV/COVID-19	Limitations of the use of ICD-coded data within disease-specific surveillance systems
Abdel-Hady (2018)	Oman	Nabdh Al Shifa (computerized online system)	N/A	N/A
Alchikh (2019)	Germany	Quality management programme at a Berlin children's hospital with the Robert Koch Institute	"ILI and (S)ARI case definitions used:<br>1. ILI_QM: fever ≥38°C with ≥ one respiratory symptom*<br>2. WHO_ILI_new: acute respiratory infection and measured fever of ≥38°C and cough with onset within the last ten days<br>3. WHO_ILI _old: fever >38° and cough or sore throat<br>4. WHO_SARI: acute respiratory infection with history of fever or measured fever of ≥38°C and cough with onset within the last ten days and requires hospitalization<br>5. CDC_ILI: fever (at least 37·8°C = 100 F) and at least one of the following: cough or sore throat and no other known cause except influenza**<br>6. ECDC_ILI: sudden onset of symptoms*** and at least one of the following: fever or feverishness or	"The infrequent use of virus-specific ICD codes may be due to physician bias, lack of familiarity, or time constraints, including assignment of codes before laboratory results are available… The usefulness of ICD codes for surveillance purposes is limited by inconsistencies and overlap between codes… ICD coding may also be influenced by seasonality (4) Expanded use of rapid diagnostics would likely increase the yield of virus-specific ICD codes"

Study	Country	Name of system	Case definition for ILI/ SARI/ ARI/ Influenza/RSV/COVID-19	Limitations of the use of ICD-coded data within disease-specific surveillance systems
			malaise or headache or muscle pain and at least one of the following: cough or sore throat or shortness of breath<br>7. ECDC_ARI: sudden onset of symptoms*** and at least one of the following: cough or sore throat or shortness of breath or coryza and a ‘clinician’s judgment that the illness is due to an infection’ ****<br>* defined as: cough, sore/red throat, coryza, apnoea, tachypnea, dyspnoea, wheezing, obstruction, chest retractions, pulmonary rales, or ear ache<br>** defined as: positive rapid test (if performed in the ED) or ICD-10 coding positive for influenza. At the same time, RSV- or Strep-A rapid tests (if performed in the ED) had to be negative and ICD-coding had to be negative for RSV<br>*** defined as: onset of symptoms <=48h prior to presentation<br>**** defined as: physician requesting participation in the QM-Program (n=789) or ordering diagnostics in	

Study	Country	Name of system	Case definition for ILI/ SARI/ ARI/ Influenza/RSV/COVID-19	Limitations of the use of ICD-coded data within disease-specific surveillance systems
			routine care (Standard-of-Care n=528), or both (n=63)"	
Amodio (2014)	Italy	The Regional Hospital Discharge Database; WHO FluNet database	N/A	"Some common limitations of all passive surveillance systems, such as underdiagnosis or deficiencies in reporting, that were different in other geographic settings may have ultimately, affected the quality of the ICD-9 CM coding in terms of completeness and accuracy in our study"
Antoon (2024)	USA	NVSN	N/A	"Estimates of influenza disease burden based on case identification with ICD-10 diagnosis codes may need to be adjusted for under ascertainment. The accuracy of ICD-10 diagnoses is associated with clinical testing. With increasing availability of influenza testing and changes in testing practices following the COVID-19 pandemic, the accuracy of influenza diagnoses may increase in the future"
Aysert-Yildiz (2019)	Turkey	GIHSN	N/A	"The diagnostic codes used in influenza surveillance are less likely to detect actual influenza cases, and studies evaluating the

Study	Country	Name of system	Case definition for ILI/ SARI/ ARI/ Influenza/RSV/COVID-19	Limitations of the use of ICD-coded data within disease-specific surveillance systems
				influenza burden using diagnostic codes might lead to false results”
Azziz-Baumgartner (2013)	Argentina	PAHO’s mortality databases and Argentina’s hospitalization databases	N/A	N/A
Bagarella (2022)	Italy	Structured and unstructured data from visits to 3 EDs of a local health assessment unit in Lombardy	N/A	N/A
Bellazzini (2011)	USA	ED syndromic surveillance system	“ILI chief complaint syndrome definition: fever, cough, headache, fatigue/malaise, muscle pain, URI symptoms, viral illness, body aches, sore throat, chills”	N/A
Bernadou (2023)	France	PMSI	N/A	N/A
Betancourt (2007)	USA	ESSENCE	N/A	“Providers may not know the coding system well and may be more likely to choose a single ICD-9 code due to time constraints”
Bhatt (2023)	USA	2019 HCUP-KID	“RSV-related hospitalizations were identified using ICD-10 codes”	“Kids Inpatient Database, which utilizes ICD-10 codes, is

Study	Country	Name of system	Case definition for ILI/ SARI/ ARI/ Influenza/RSV/COVID-19	Limitations of the use of ICD-coded data within disease-specific surveillance systems
				retrospective cross-section data created using discharge abstracts by hospitals for billing, which is subject to administrative errors”
Blatt (2024)	USA	EHR data from four health care systems: SUNY Health System; Duke University Health System; Renown Children's Hospital; and Tampa General Hospital/ University of South Florida	“Infants were classified as having ‘RSV bronchiolitis’ if they had a positive RSV test (antigen and/or PCR) and an encounter with one of the corresponding ICD-10 codes (B97.4, J12.1, J20.5, J21.0, J21.8, or J21.9)”	N/A
Boender (2022)	Germany	The AKTIN Emergency Department Data Registry and the ESEG project	“Case definitions for acute respiratory infection (ARI), severe acute respiratory infection (SARI), influenza-like illness (ILI), respiratory syncytial virus infection (RSV) and COVID-19 were based on a combination of ICD-10 codes, and/or chief complaints, sometimes combined with information on hospitalisation and age”	“ICD-10 codes for COVID-19 diagnosis were not available from the beginning of the pandemic, leading to underreporting of the true number of COVID-19 cases”
Bouckaert (2023)	Belgium	B-HDDS	“RSV-associated hospital episodes were identified by the following principal or secondary ICD-10-CM	“ICD-10-CM codes were used to identify RSV-associated hospitalizations, which are not

Study	Country	Name of system	Case definition for ILI/ SARI/ ARI/ Influenza/RSV/COVID-19	Limitations of the use of ICD-coded data within disease-specific surveillance systems
			diagnosis codes: B974 (RSV as the cause of diseases classified elsewhere), J121 (pneumonia due to RSV), J205 (acute bronchitis due to RSV) and J210 (acute bronchiolitis due to RSV)"	necessarily based on laboratory tests"
Bouzille (2018)	France	eHOP clinical data warehouse technology of the academic hospital of Rennes	"Our definition of ILI case covered any patient visit for which a document that matched a given query was generated (ICD-10 query: DRGs having at least one code belonging to the influenza-related ICD-10 chapters: J09.x, J10.x or J11.x; Biology query: We relied on the local terminology used by the laboratory information system to retrieve all flu PCR test results (negative and positive))"	N/A
Bruzda (2021)	USA, Dominican Republic, El Salvador, Honduras	CREDO system	World Health Organization's 2011 revised case definitions for Influenza Like Illness (ILI).	NA
Buda (2017)	Germany	Hospitals belonging to the HELIOS Kliniken Gmb	"Three different SARI case definitions (CD) based on ICD-10 codes for discharge diagnoses of respiratory tract infections (J09 - J22): basic CD (BCD), using only primary diagnoses;	N/A

Study	Country	Name of system	Case definition for ILI/ SARI/ ARI/ Influenza/RSV/COVID-19	Limitations of the use of ICD-coded data within disease-specific surveillance systems
			sensitive CD (SCD), using primary and secondary diagnoses; timely CD (TCD), using only primary diagnoses of patients hospitalized up to one week".	
Cai (2020a)	Germany	SEEDARE; ICOSARI; and from the virological surveillance at the Robert Koch Institute	"We defined an RSV-ICD-case based on SEEDARE data as a medical consultation with any of the three RSV-specific ICD-10 code diagnoses (J12.1 RSV pneumonia, J20.5 acute bronchitis due to RSV, and J21.0 acute bronchiolitis due to RSV).6 We defined a RSV-ICD-case based on ICOSARI data as a hospitalization with any of the three RSV-specific ICD-10 code diagnoses as primary discharge diagnosis. In the virological surveillance, we defined a confirmed-RSV-case as a by real-time reverse transcriptase polymerase chain reaction (rtRT-PCR) confirmed RSV sample"	"RSV-specific ICD-10 codes underestimate the number of actual RSV infections… The RSV coding behavior of physicians in primary care may vary during and out of RSV season, based on use of laboratory diagnostics, age of patient, and level of coding awareness. The differences in coding behavior may lead to information bias"
Cai (2020b)	Germany	ICOSARI database	"• Severe ARI (SARI) case: any patient hospitalized with any of the ARI ICD-10 codes J09-J22 (J09-J11: influenza, J12-J18: pneumonia, J20: acute bronchitis, J21: acute	"ICOSARI is a ICD-10-based syndromic surveillance system. Virological data of the ICOSARI network were not available. As the coding behavior of physicians may

Study	Country	Name of system	Case definition for ILI/ SARI/ ARI/ Influenza/RSV/COVID-19	Limitations of the use of ICD-coded data within disease-specific surveillance systems
			bronchiolitis, J22: unspecified acute lower respiratory infection) as primary or secondary discharge diagnosis13,15; If a patient was readmitted to hospital, the patient would be counted again.<br>• RSV case: SARI case diagnosed with any of the RSV-specific ICD-10 codes (J12.1: RSV pneumonia, J20.5: acute bronchitis due to RSV, J21.0: acute bronchiolitis due to RSV) as primary or secondary discharge diagnosis"	vary based on use of laboratory diagnostics and level of coding awareness, some true cases may be missed or wrongly included due to miscoding which could lead to information bias"
Chow (2020)	USA	The US FluSurv-NET	"We defined laboratory-confirmed influenza virus infection within 14 days before or 3 days or less after hospital admission based on a positive result of reverse transcription–polymerase<br>chain reaction, rapid antigen assay, direct or indirect fluorescent staining, or viral culture. Acute respiratory or non-respiratory diagnoses were defined using ICD-9-CM and ICD-10 discharge diagnosis codes"	"We further found that using pneumonia or influenza ICD codes failed to capture 14% of patients with laboratory-confirmed influenza reported to FluSurvNET. These findings have implications for the potential underestimation of rates in studies that identify influenza using administrative data… We identified acute diagnoses by ICD codes, a method commonly used to classify patient diagnoses in several studies of influenza complications, which may have inherent limitations that led to misclassification"

Study	Country	Name of system	Case definition for ILI/ SARI/ ARI/ Influenza/RSV/COVID-19	Limitations of the use of ICD-coded data within disease-specific surveillance systems
Cocchio (2023)	Italy	Hospital discharge records of Veneto's database	RSV-related diseases was defined as "hospitalizations with at least one of the following ICD9-CM codes: 079.6—Respiratory Syncytial Virus; 466.11—acute bronchiolitis due to RSV; and 480.1—pneumonia due to RSV".	"By using ICD-9 codes, many factors can affect the data during their generation; among them, the most important one is the lack of the application of testing in older children and adults"
Cocoros (2023)	USA	ESP public health surveillance platform	"COVID-19–like illness (CLI) is defined as occurrence of a diagnosis code for lower respiratory tract infection and a fever or a diagnosis code for an upper respiratory infection and a fever"	"While our work provided insight into COVID-19 activity without dependence on laboratory testing, it was not necessarily specific to SARS-CoV-2 and may have detected syndromes caused by other respiratory viruses. The algorithm's performance will need to be monitored over time"
Eick-Cost (2022)	USA	DMSS	"The case definitions were developed using interim clinical case definitions proposed by the CDC and expert knowledge, which incorporated random chart reviews of cases to better ascertain coding practices of DOD physicians. The COVID-19 case definition (ICD-10-CM: U07.1); The COVID-specific case definition (10 additional codes added); The COVID-like illness case definition (19	"The analysis required a medical encounter, so asymptomatic and non-medically attended individuals will not be captured in this analysis and should be considered a gap in surveillance utilizing ICD-10-CM case definitions"

Study	Country	Name of system	Case definition for ILI/ SARI/ ARI/ Influenza/RSV/COVID-19	Limitations of the use of ICD-coded data within disease-specific surveillance systems
			additional codes added to the COVID-specific definition)”	
Elkin (2016)	USA	Biosense biosurveillance system	“The CDC defines an influenza-like illness as fever plus either cough and/or sore throat in the absence of a known cause other than influenza”	“Respiratory Syncytial Virus testing was done very uncommonly and may account for some portion of the ICD9-CM codes for Influenza that tested negative”
Farah (2023)	Lebanon	SARI sentinel surveillance system; and the Ministry of Public Health’s hospital billing database	“SARI cases were defined as acute respiratory infection patients with a history of fever or measured fever of $\geq 38^{O}$C and cough with onset of symptoms within the last 10 days and requiring hospitalization”	“The selection of respiratory cases from the ICD-10 codes related to influenza and pneumonia (J09-J18) only could have underestimated the real burden of influenza as some cases might have been coded under J00-J99 ICD-10 codes or might be other consequences of the disease like cardiovascular events and exacerbation of comorbidities among others, which should not be ignored”
Feemster (2016)	USA	EHRs from a large paediatric network of clinics	“Healthcare-associated influenza-like illness (HA-ILI) cases were defined as ILI in children who returned for an ILI visit within 24 hours to 8 days after a non-ILI related clinic visit. Non-ILI cases were defined as no documented ILI within 8 days after a non-ILI clinic visit”	“Our HA-ILI definition (based on ICD codes) was syndromic, and we were unable to confirm new acquisition of a respiratory pathogen between the initial visit and subsequent visit”

Study	Country	Name of system	Case definition for ILI/ SARI/ ARI/ Influenza/RSV/COVID-19	Limitations of the use of ICD-coded data within disease-specific surveillance systems
Fung (2023)	USA	CMS Virtual Research Data Center	"Long COVID was identified by (a) the designated long COVID code B94.8 (code-based definition), or (b) any of 11 symptoms identified in the WHO definition (symptom-based definition), from 1 to 3 months post-infection"	"Relying on specific long COVID diagnostic codes results in significant underreporting"
Gerbier-Colomban (2014)	France	UrgIndex surveillance system at the North Hospital Group of the Lyon University Hospitals	N/A	N/A
Giordani (2024)	Italy	Hospital discharge records (collected by Italian MoH), linked with National Tax Registry	N/A	"Due to the lack of specified COVID-19 codes from the onset of the pandemic, the overall hospitalizations detected through the algorithm might be underestimated"
Girit (2017)	Turkey	GIHSN	"The European Centre for Disease Prevention and Control (ECDC) define ILI as; sudden onset of symptoms and at least one of the following: i) fever or feverishness, ii) malaise, iii) headache, iv) myalgia and at least one of the following three respiratory symptoms: i) cough, ii) sore throat, iii) shortness of breath"	N/A

Study	Country	Name of system	Case definition for ILI/ SARI/ ARI/ Influenza/RSV/COVID-19	Limitations of the use of ICD-coded data within disease-specific surveillance systems
Gundlapalli (2021)	USA	Death certificates from CDC	N/A	N/A
Habbous (2023)	Canada	DAD and NACRS	"The International Classification of Diseases, 10th revision (ICD-10) codes were used to identify influenza (J09, J10.0, J10.1, J10.8, J11.0, J11.1, J11.8) and RSV (J12.1, J20.5, J21.0, B97.4)"	"While ICD-10 coding has moderate sensitivity (73% for influenza and 69% for RSV), provincial systemic laboratory reporting to confirm respiratory virus diagnoses (e.g. OLIS) was unavailable for this dataset"
Hagiwara (2022)	Japan	RWD database	"We defined patients with clinically diagnosed influenza (CDI) based on ICD-10 administrative codes (J09 to J18 and J22), which are associated with influenza and pneumonia. Any of these codes appearing at any position in the patient's discharge diagnosis list was captured. In addition, we defined patients with laboratory-confirmed influenza (LCI) based on positive influenza test results (including influenza rapid diagnostic test [IRDTs] and reverse transcription polymerase chain reaction [RT-PCR])"	N/A
Ishiguro (2024)	Japan	Claims data from the National Center for Global Health and Medicine Hospital	N/A	N/A

Study	Country	Name of system	Case definition for ILI/ SARI/ ARI/ Influenza/RSV/COVID-19	Limitations of the use of ICD-coded data within disease-specific surveillance systems
Johnson (2022)	USA	Biobank at CCPM	“Participants who received an ICD-10 diagnosis code of U07.1 or at least 1 of 11 COVID-19-specific encounter primary diagnoses were considered an ‘EHR-confirmed case’ for COVID-19”	N/A
Keck (2014)	USA	The Indian Health Service’s Influenza Awareness System	N/A	N/A
Khanh (2021)	Vietnam	Vietnam's SARI sentinel surveillance system; Vietnam's MoH EMRs system	“•We defined an ARI hospitalization as a hospitalization with an admission code from the ICD-10, for either acute upper respiratory infection (J06) or codes to approximate SARI (influenza, J09–11; pneumonia, J12–18; or other acute lower respiratory infections, J20–J22).<br>•We defined WHO SARI case definition as sudden onset of fever >38°C, with cough or sore throat, and with shortness of breath or difficulty breathing, and illness requiring hospitalization; the revised 2014 definition was temperature of >38°C or history of fever, cough of duration <10 days, and illness requiring hospitalization”	N/A

Study	Country	Name of system	Case definition for ILI/ SARI/ ARI/ Influenza/RSV/COVID-19	Limitations of the use of ICD-coded data within disease-specific surveillance systems
Leiner (2023)	Germany	IQM network	"SARI cases were defined by ICD-codes J09-J22 and COVID-19 by ICD-codes U07.1 and U07.2"	N/A
Light (2008)	USA	Florida Department of Health's epidemiology surveillance network	"RSV was considered present when 10% of laboratory tests were positive in a given month and the duration of seasons was determined by the number of consecutive months threshold values were exceeded. Hospitalizations in children 24 months of age and younger were defined as RSV related if any of the following RSV-specific ICD-9-CM codes appeared on the discharge summary: 079.6 RSV; 466.11 acute bronchiolitis caused by RSV; and 480.1 pneumonia caused by RSV"	"The Florida Department of Health does not provide confirmation on the RSV laboratory testing methods used in all cases, and the fewest number of tests were performed during shoulder months when there is an increased chance for false-positive results"
Loubet (2024)	France	PMSI	"An episode of RSV was defined to qualify patients presenting with either a single RSV-related stay or with additional subsequent RSV-related stays within a 90-day period. Any stay after 90 days of the index date was considered as a new episode"	"There is so far limited routine diagnostic testing for respiratory viruses in France and laboratory results are not recorded in the PMSI making infections diagnoses only identifiable through ICD-10 codes. This situation may lead to an important under-reporting of RSV episodes especially in the elderly population. Thus, underestimation

Study	Country	Name of system	Case definition for ILI/ SARI/ ARI/ Influenza/RSV/COVID-19	Limitations of the use of ICD-coded data within disease-specific surveillance systems
				of the disease burden of RSV is likely to occur in studies relying on discharge codification and differences in coding behavior may also lead to information bias”
Mad Tahir (2023)	Malaysia	MY-DRG Casemix database (of a teaching hospital in Malaysia)	Influenza and its complications were defined as “cases with principal or secondary diagnoses coded in the ICD-10 as J09, J10.0, J10.1, J10.8, J11.0, J11.1, J11.8, J12.8, and J12.9”	N/A
Marsden-Haug (2007)	USA	An automated ILI surveillance report incorporated into ESSENCE	“Clinical case definition of ILI is a fever >100.5°F (38°C) and either a cough or sore throat”	N/A
Matias (2017)	USA	Hospitalisation data from US Nationwide Inpatient Sample; and virological data from FluView	N/A	“Risk status could only be determined by the presence or absence of ICD codes during a single hospitalization episode, and assignment of risk status critically depended upon whether the physician had mentioned among the discharge diagnoses any existing underlying disease. In clinical practice, it is likely that the physician would fail to mention some underlying diseases or factors that are categorized as high risk.

Study	Country	Name of system	Case definition for ILI/ SARI/ ARI/ Influenza/RSV/COVID-19	Limitations of the use of ICD-coded data within disease-specific surveillance systems
				This may have led to misassignment of risk status. In contrast, symptoms that were part of the influenza illness may have been listed as underlying disease, leading to overestimation of risk”
Mattiuzzi (2023)	USA	CDC WONDER Online Database	N/A	N/A
McLeod (2009)	New Zealand	Wellington ED Respiratory Syndromic Surveillance System	“Respiratory syndrome was defined as having a primary diagnosis of one of 68 ICD 10 codes selected for the purposes of this pilot study. The ICD 10 code selections were validated through comparisons with existing syndrome definitions and codes, existing surveillance data (General Practice Influenza Sentinel Surveillance), and local and national hospitalisation data”	N/A
McMurry (2024)	USA	an open-source AI–based natural language processing pipeline that includes a large language model	“A diagnosis of COVID-19 was defined as a positive SARS-CoV-2 polymerase chain reaction (PCR) test or the presence of ICD-10 code U07.1 for COVID-19 during the same ED encounter in which symptoms were evaluated. COVID-19 symptoms were defined based on Centers for Disease	“Natural language processing (NLP) was more sensitive than ICD-10 codes in identifying symptoms, and some symptoms could only be detected using NLP”

Study	Country	Name of system	Case definition for ILI/ SARI/ ARI/ Influenza/RSV/COVID-19	Limitations of the use of ICD-coded data within disease-specific surveillance systems
			Control and Prevention (CDC) criteria—fever or chills, cough, shortness of breath or difficulty breathing, fatigue, muscle or body aches, headache, new loss of taste or smell, sore throat, congestion or runny nose, nausea or vomiting, and diarrhea"	
Milliren (2023)	USA	Paediatric Hospital Information System	"COVID-19 was identified by primary or secondary ICD-10 diagnosis code U07.1"	"We were unable to discern admissions specifically for COVID-related symptoms given the limitations of billed diagnosis codes and administrative data".
Mira-Iglesias (2022)	Spain	VAHNSI	"RSV-associated disease was defined based on the following ICD-10 codes recorded at hospital discharge: Lower Respiratory Infection (LRI): J09-J22, bronchiolitis: J21 and pneumonia: J12-J18"	N/A
Moore (2011)	Australia	SynSurv	Influenza case is defined as "laboratory confirmed influenza cases notified to the Department of Health".	"Case diagnosis via ICD codes is influenced by what ED staff believes to be occurring in the community"
Motlogeloa (2023)	South Africa	Data from the South African Medical Research Council Respiratory and	"ARI diseases are defined as infections of the respiratory system caused by viruses or bacteria that develop in less than fifteen days and show symptoms	N/A

Study	Country	Name of system	Case definition for ILI/ SARI/ ARI/ Influenza/RSV/COVID-19	Limitations of the use of ICD-coded data within disease-specific surveillance systems
		Meningeal Pathogens Research Unit database; and the Discover Medical Insurance Scheme claims database	such as coughing, nasal congestion, airway obstruction, sore throat, dysphonia, or respiratory distress, whether they are accompanied by fever. The conditions under this wide classification that are the subject of this study include influenza, respiratory syncytial virus (RSV), acute nasopharyngitis, influenza with pneumonia, influenza with other respiratory manifestations, and influenza with other features".	
Moura (2024)	Canada	CCEDRRN registry linked to administrative diagnostic codes	"We defined lab-confirmed SARS-CoV-2 based on at least one positive PCR in the 0–14 days before the ED presentation and/or during hospitalization (in those admitted from ED)"	"Administrative health data diagnostic codes were less sensitive for identifying lab-confirmed SARS-CoV-2 in patients discharged from the ED. This limitation is important to acknowledge if ICD code U07.1 is used for SARS-CoV-2 case detection, for research and public health purposes"
Murray (2023)	Australia	NATA approved, batch testing, in-house real-time PCR platform	"The hospital coding for each admission was evaluated for diagnoses where the influenza virus was identified (ICD-10 code J09 or J10) or suspected (ICD-10 code J11)". PCR detection of influenza	"Hospital administrative coding for influenza may underestimate the true incidence of influenza-associated admissions"

Study	Country	Name of system	Case definition for ILI/ SARI/ ARI/ Influenza/RSV/COVID-19	Limitations of the use of ICD-coded data within disease-specific surveillance systems
			was used as the 'gold-standard' for diagnosis.	
Ortiz (2014)	USA	State Inpatient Databases from Arizona, California, and Washington; and Regional influenza surveillance data from CDC	"Critical illness hospitalizations were defined as any hospitalizations with ICD-9-CM diagnosis or procedure codes for acute respiratory failure (96.7 and any of 518.5, 518.81, or 518.82), severe sepsis (995.92, 785.52, or any codes for infection and organ dysfunction), or in-hospital death"	"Surveillance studies which only consider hospitalizations that include a diagnosis of pneumonia may underestimate the total burden of influenza hospitalizations"
Pattie (2009)	USA	AHLTA	"The Centers for Disease Control and Prevention (CDC) defines influenza-like illness (ILI) for its sentinel providers as fever (temperature ≥100.5◦F or 37.8◦C) and a cough and/or a sore throat in the absence of a known cause other than influenza"	N/A
Pumarola (2023)	Spain	PHDB for Spain	The European Union's definition of influenza; being close to the WHO's definition of ILI	"The study is based on extrapolated administrative data, and subject to coding errors or missing information. Thus, administrative data without a systematic testing for influenza would lead to under-detection of influenza, thus underestimating the impact of influenza virus… The study period has included two ICD systems, but

Study	Country	Name of system	Case definition for ILI/ SARI/ ARI/ Influenza/RSV/COVID-19	Limitations of the use of ICD-coded data within disease-specific surveillance systems
				ICD-10 brought a greater granularity, and it may have impacted coding practices”
Ramos-Rincon (2024)	Spain	SNSSHD, specifically the Hospital Care Activity Record - Minimum Basic Data Set (Registro de actividades especializadas- Conjunto Mínimo Básico de datos)	“Hospitalization related to influenza (SARI) was defined as all recorded hospital admissions with a diagnosis of influenza, defined by ICD-10 codes J09, J10, J11, as either a primary or secondary diagnosis”	“Limitations include those typically associated with the use of medico-administrative databases with coding rules. First, there are some risks of bias, related to: underreporting, severity (the most severe cases are those that are admitted, entailing an overrepresentation of mortality), diagnostic accuracy (the diagnosis of influenza can be clinical or virological by RT-PCR, and clinically diagnosed cases may be other respiratory viral infections)… Moreover, we have incorporated ICD-10 codes pertaining to influenza as either a primary or secondary diagnosis. Notably, instances where influenza is classified as a secondary diagnosis may result in an overestimation of metrics such as length of hospital stay, admission to the intensive care unit (ICU), mortality rates, and

**Study**	**Country**	**Name of system**	**Case definition for ILI/ SARI/ ARI/ Influenza/RSV/COVID-19**	**Limitations of the use of ICD-coded data within disease-specific surveillance systems**
				associated costs, in comparison to cases where it is designated as a primary diagnosis”
Ramsay (2010)	Australia	Community-based Victorian GP surveillance program	“Definition of an ILI (fever, cough and malaise), as used by the general practice sentinel surveillance program”	“Syndromic surveillance of a range of ICD-10 codes was not a sensitive method to identify patients admitted with an ILI”
Reed (2014)	USA	EIP Influenza Surveillance Network	“Laboratory testing for influenza is ordered at the discretion of clinicians providing medical care, and confirmation may include a positive result from viral culture, direct or indirect fluorescent antibody staining, rapid antigen test, reverse transcription polymerase chain reaction (PCR), or documentation of a positive test result in a patient’s medical record”	“In this study population, approximately 5% of patients did not have any respiratory ICD-9 code, and about 12% of patients with 1 of the included complications did not have a respiratory complication. These complications may be underrepresented if physicians are less likely to test such patients for influenza”
Ricoca Peixoto (2023)	Portugal	Clinical registries of Portuguese NHS hospitals	“A confirmed case of COVID-19 is anyone with a positive RT-PCR result for SARS-CoV-2 RNA in nasopharyngeal and/or oropharyngeal specimens regardless of clinical or epidemiological criteria”	“Using Elixhauser comorbidity Index categories to reduce arbitrariness in categorizing ICD-10 comorbidities may include a range of clinical entities and clinical severities in some groups, that may include milder comorbidities, possibly underestimating risk for

Study	Country	Name of system	Case definition for ILI/ SARI/ ARI/ Influenza/RSV/COVID-19	Limitations of the use of ICD-coded data within disease-specific surveillance systems
				more specific comorbidities within those categories”
Saleh (2024)	USA	IDR i2b2 platform	N/A	N/A
San-Roman-Montero (2019)	Spain	National Surveillance System for Hospital Data (Conjunto Mínimo Básico de Datos)	N/A	N/A
Schirmer (2010)	USA	ESSENCE	“Visits were counted as ILI if they had at least one ICD-9-CM diagnostic code included in the 31 codes that define the ILI syndrome group but these do not necessarily represent laboratory confirmed influenza cases”	“Limited identification of confirmed cases since confirmatory testing is no longer indicated for many patients with suspected pandemic H1N1 2009… Another limitation with the current VA ESSENCE system is the reliance on ICD-9-CM codes. The codes are not assigned until the end of the visit and can take several days to be coded since in general VA provider reimbursement is not directly linked with coding of patient visits… Accuracy of ICD-9-CM codes can be variable”
Shappell (2023)	USA	Mass General Brigham healthcare System	N/A	“Because our medical record reviews were conducted among PCR-positive hospitalizations, the true positive

Study	Country	Name of system	Case definition for ILI/ SARI/ ARI/ Influenza/RSV/COVID-19	Limitations of the use of ICD-coded data within disease-specific surveillance systems
				predictive value of ICD-10 codes might be even lower. We advise caution when interpreting studies which identify COVID-19 hospitalizations using ICD-10 codes during the current era"
Sigmundsdottir (2010)	Iceland	CHS-CDC	"The European Union's case definitions for ILI, confirmed cases of seasonal influenza and confirmed cases of 2009 pandemic influenza A(H1N1) were used"	"It is possible that physicians were affected by the introduction of a new reporting system (including ICD coding) and the ongoing pandemic in their clinical assessment"
Sivakumaran (2023)	Wales	SAIL Databank	N/A	"We demonstrate how using ICD-10 diagnosis codes to detect respiratory pathogens associated with COPD admissions in our study would have considerably underestimated the burden of these infections. Researchers using ICD-10 codes as substitutes for raw microbiology data should be aware that coding deficiencies may lead to a substantial loss in accuracy. Other studies have also identified underutilization of diagnostic microbiology testing and low sensitivity of ICD codes to identify

Study	Country	Name of system	Case definition for ILI/ SARI/ ARI/ Influenza/RSV/COVID-19	Limitations of the use of ICD-coded data within disease-specific surveillance systems
				laboratory-confirmed non-influenza respiratory infection”
Sotomayor (2018)	Chile	SARI sentinel surveillance database; Records of hospital discharges and national deaths, managed by the DSHI of the MoH of Chile	“SARI case is defined as a patient who is hospitalized with fever or history of fever of 38°C or higher, cough, and respiratory distress (i.e., polypnea and decreased oxygen saturation)”	“First, we used a proxy to quantify the number of severe influenza cases—the hospital discharges or deaths due to pneumonia and influenza at the national level, and the application of the surveillance percent positivity for influenza from six hospitals to these cases, which might under-estimate the mortality rates, especially in the extreme age-groups, where the suspicion of influenza is low in comparison with other etiologies for the death. Also, analyzing exclusively hospital discharges due to pneumonia and influenza, excluding those cases that had chronic diseases in which the influenza infection triggered the hospitalization, or those cases where the clinical pattern is not a typical flu or pneumonia. Further analysis must be performed with a more sensitive definition like ICD 10 J00 to J99”

Study	Country	Name of system	Case definition for ILI/ SARI/ ARI/ Influenza/RSV/COVID-19	Limitations of the use of ICD-coded data within disease-specific surveillance systems
Taylor (2016)	UK	CPRD; HES database; ONS database; UK national surveillance system at PHE	N/A	"Changes in coding practices or in the quality of diagnoses recorded in the databases over time could not be accounted for in our model"
Thiam (2022)	France	SurSaUD®	COVID-19 case is defined "based on WHO coding recommendations for COVID-19"	"Data relating to specific COVID-19 health care measures were not systematically entered into the system, and thus, the number of ED visits may thus have been underestimated… Possible bias in the identification of COVID-19-related visits due to miscoded visits"
Torres (2023)	Portugal	SARI sentinel surveillance system	"SARI cases were defined as patients containing ICD-10 codes for influenza-like illness, cardiovascular diagnosis, respiratory diagnosis, and respiratory infection in their primary admission diagnosis"	"Using only primary diagnoses could capture mainly the population without major comorbidities, in particular, in hospitalizations due to influenza-like illness. SARI cases with underlying chronic disease (e.g., cardiovascular diseases) may have their comorbidity coded in the primary diagnosis and ICD-10 codes corresponding to symptoms of influenza-like illness in their secondary diagnosis, as the exacerbation of the chronic disease could play a major role in their

Study	Country	Name of system	Case definition for ILI/ SARI/ ARI/ Influenza/RSV/COVID-19	Limitations of the use of ICD-coded data within disease-specific surveillance systems
				hospitalization. This is in line with the increase in hospitalizations due to cardiovascular diagnosis during the epidemic activity of Influenza in Portugal (between March and May 2022)”
Trucchi (2019)	Italy	Syndromic Surveillance System	“ILI case was defined as:<br>• statement by physician: patient with “suspected or confirmed Influenza” or “ILI”;<br>• presence of fever and respiratory symptoms (sore throat OR cough OR phlogosis/inflammation of respiratory tract OR ...) recorded during the ED access;<br>• symptoms reported by the patient within 7 days: fever and respiratory symptoms (sore throat OR cough OR phlogosis/inflammation of respiratory tract OR of ...);<br>• presence of dyspnea or respiratory failure preceded by ILI OR fever and respiratory symptoms; even if the access is due to potential non-infectious complications of influenza”	N/A
Wan Puteh (2023)	Malaysia	Casemix system	“ILI is defined based on the diseases history of cough and fever measured at	“The influenza data used in the study relied on the ICD-10 codes

Study	Country	Name of system	Case definition for ILI/ SARI/ ARI/ Influenza/RSV/COVID-19	Limitations of the use of ICD-coded data within disease-specific surveillance systems
			≥ 38oC. SARI is measured as fever of ≥ 38oC; with cough onset within the last ten (10) days of the incubation period”	that were approved by experts, to reflect influenza disease burden in Malaysia”
Wan Puteh (2024)	Malaysia	Casemix system	“ILI cases were identified based on the WHO case definition for ILI, which includes individuals presenting acute respiratory infection with measured fever of □38˚C and cough, with onset within the last ten days. On the other hand, SARI cases were identified using the WHO case definition for SARI, which encompasses individuals with an acute respiratory infection, presenting with a history of fever or measured fever of 38˚C, and cough, with onset within the last ten days, and a requirement for hospitalization due to the severity of their condition”	“The influenza data used in the study relied on the ICD-10 codes that were approved by experts, to reflect influenza disease burden in Malaysia”
Wander (2023)	USA	US Department of VA’s Corporate Data Warehouse and CSDR	N/A	“The U09.9 code is a proxy for the presence of pandemic, post–COVID-19 condition (PCC) and codes may be used inconsistently especially in the early adoption period or may be underused in populations with limited access to care”

Study	Country	Name of system	Case definition for ILI/ SARI/ ARI/ Influenza/RSV/COVID-19	Limitations of the use of ICD-coded data within disease-specific surveillance systems
Wells (2022)	Scotland	SARI surveillance system: using data from SMR01 and RAPID	“Three SARI case definitions (CDs) were used:<br>1. Sensitive case definition (CD1): Any patient discharged from a Scottish hospital who had at least one of the specified ICD-10 codes listed in any of the six patient diagnosis fields in their General Acute Inpatient and Day Case - Scottish Morbidity Record (SMR01) for each individual hospital stay.<br>2. Specific case definition (CD2): Any patient discharged from a Scottish hospital who had one of the specified ICD-10 codes listed in the main/first patient diagnosis field in their General Acute Inpatient and Day Case - Scottish Morbidity Record (SMR01) for each individual hospital stay.<br>3. Timely case definition (CD3): Any patient admitted to a Scottish hospital who had at least one of the specified ICD-10 codes listed in any of the six patient diagnosis fields in their Rapid Preliminary Inpatient Dataset (RAPID) records for each individual hospital stay”	“The validation highlighted several periods of poor completion of ICD-10 codes within the RAPID data set”

Study	Country	Name of system	Case definition for ILI/ SARI/ ARI/ Influenza/RSV/COVID-19	Limitations of the use of ICD-coded data within disease-specific surveillance systems
Whittaker (2023)	Norway	NPR and NoPaR	N/A	"Different ICD-10 code combinations were registered among patients in both main cause categories. This highlights that clinicians may assess the main cause of admission differently for patients with similar diagnostic codes, leading to non-differential misclassification. Also, we did not have access to full ICD-10 codes for all diagnostic categories, which limited the exploration of whether more detailed code combinations could more precisely represent persons hospitalized due to COVID-19. We also only considered the distribution of ICD-10 codes in this analysis. However, other parameters could additionally inform more precise proxies"
Wick (2023)	Germany	German Institute for the Hospital Remuneration System	"RSV-associated hospitalization cases were defined using the 10th revision of the International Classification of Diseases (ICD-10), that is, the RSV-specific codes J12.1 (RSV pneumonia), J20.5 (acute bronchitis due to RSV), and J21.0 (acute	"We excluded cases with only secondary diagnoses of RSV-specific ICD-10 codes to avoid double counting and to exclude cases that were primarily hospitalized for other conditions. Therefore, we could not make use

Study	Country	Name of system	Case definition for ILI/ SARI/ ARI/ Influenza/RSV/COVID-19	Limitations of the use of ICD-coded data within disease-specific surveillance systems
			bronchiolitis due to RSV), when coded as principal diagnosis at hospital discharge. SARI was defined as hospitalization cases with a principal diagnosis of any ICD-10 code of J09–J22 (J09–J11: influenza, J12–J18: pneumonia, J20: acute bronchitis, J21: acute bronchiolitis, and J22: unspecified acute lower respiratory infection) at hospital discharge"	of the ICD-10 code B97.4 (RSV as the cause of diseases classified to other chapters), although we do not expect considerable underestimation of hospitalization incidence"
Widgren (2010)	Denmark	Danish National Patient Registry	"Influenza-associated hospitalizations refers to records of hospital visits/ admissions stated as in-patient hospitalizations, with either of the selected ICD-10 codes as a primary or secondary diagnosis extracted from the registry and included in the analysis"	"The main limitation to this surveillance system turned out to be the delay in data delivery. Hence, the number of influenza-associated hospitalizations in the current season could increase as we receive updates of the registry"

PRISMA 2020 checklist

Section and Topic	Item #	Checklist item	Location where item is reported
**TITLE**			
Title	1	Identify the report as a systematic review.	1
**ABSTRACT**			
Abstract	2	See the PRISMA 2020 for Abstracts checklist.	2-3
**INTRODUCTION**			
Rationale	3	Describe the rationale for the review in the context of existing knowledge.	3-5
Objectives	4	Provide an explicit statement of the objective(s) or question(s) the review addresses.	5
**METHODS**			
Eligibility criteria	5	Specify the inclusion and exclusion criteria for the review and how studies were grouped for the syntheses.	6
Information sources	6	Specify all databases, registers, websites, organisations, reference lists and other sources searched or consulted to identify studies. Specify the date when each source was last searched or consulted.	5
Search strategy	7	Present the full search strategies for all databases, registers and websites, including any filters and limits used.	52-54
Selection process	8	Specify the methods used to decide whether a study met the inclusion criteria of the review, including how many reviewers screened each record and each report retrieved, whether they worked independently, and if applicable, details of automation tools used in the process.	5-6
Data collection process	9	Specify the methods used to collect data from reports, including how many reviewers collected data from each report, whether they worked independently, any processes for obtaining or confirming data from study investigators, and if applicable, details of automation tools used in the process.	6
Data items	10a	List and define all outcomes for which data were sought. Specify whether all results that were compatible with each outcome domain in each study were sought (e.g. for all measures, time points, analyses), and if not, the methods used to decide which results to collect.	6
	10b	List and define all other variables for which data were sought (e.g. participant and intervention characteristics,	6

Section and Topic	Item #	Checklist item	Location where item is reported
		funding sources). Describe any assumptions made about any missing or unclear information.	
Study risk of bias assessment	11	Specify the methods used to assess risk of bias in the included studies, including details of the tool(s) used, how many reviewers assessed each study and whether they worked independently, and if applicable, details of automation tools used in the process.	6-7
Effect measures	12	Specify for each outcome the effect measure(s) (e.g. risk ratio, mean difference) used in the synthesis or presentation of results.	N/A
Synthesis methods	13a	Describe the processes used to decide which studies were eligible for each synthesis (e.g. tabulating the study intervention characteristics and comparing against the planned groups for each synthesis (item #5)).	7
	13b	Describe any methods required to prepare the data for presentation or synthesis, such as handling of missing summary statistics, or data conversions.	N/A
	13c	Describe any methods used to tabulate or visually display results of individual studies and syntheses.	6-7
	13d	Describe any methods used to synthesize results and provide a rationale for the choice(s). If meta-analysis was performed, describe the model(s), method(s) to identify the presence and extent of statistical heterogeneity, and software package(s) used.	7
	13e	Describe any methods used to explore possible causes of heterogeneity among study results (e.g. subgroup analysis, meta-regression).	N/A
	13f	Describe any sensitivity analyses conducted to assess robustness of the synthesized results.	N/A
Reporting bias assessment	14	Describe any methods used to assess risk of bias due to missing results in a synthesis (arising from reporting biases).	N/A
Certainty assessment	15	Describe any methods used to assess certainty (or confidence) in the body of evidence for an outcome.	N/A
**RESULTS**			
Study selection	16a	Describe the results of the search and selection process, from the number of records identified in the search to the number of studies included in the review, ideally using a flow diagram.	7-8
	16b	Cite studies that might appear to meet the inclusion criteria, but which were excluded, and explain why they	N/A

Section and Topic	Item #	Checklist item	Location where item is reported
		were excluded.	
Study characteristics	17	Cite each included study and present its characteristics.	55-73
Risk of bias in studies	18	Present assessments of risk of bias for each included study.	74-84
Results of individual studies	19	For all outcomes, present, for each study: (a) summary statistics for each group (where appropriate) and (b) an effect estimate and its precision (e.g. confidence/credible interval), ideally using structured tables or plots.	9; 85-145
Results of syntheses	20a	For each synthesis, briefly summarise the characteristics and risk of bias among contributing studies.	9-34
	20b	Present results of all statistical syntheses conducted. If meta-analysis was done, present for each the summary estimate and its precision (e.g. confidence/credible interval) and measures of statistical heterogeneity. If comparing groups, describe the direction of the effect.	N/A
	20c	Present results of all investigations of possible causes of heterogeneity among study results.	N/A
	20d	Present results of all sensitivity analyses conducted to assess the robustness of the synthesized results.	N/A
Reporting biases	21	Present assessments of risk of bias due to missing results (arising from reporting biases) for each synthesis assessed.	N/A
Certainty of evidence	22	Present assessments of certainty (or confidence) in the body of evidence for each outcome assessed.	N/A
**DISCUSSION**			
Discussion	23a	Provide a general interpretation of the results in the context of other evidence.	34-37
	23b	Discuss any limitations of the evidence included in the review.	38
	23c	Discuss any limitations of the review processes used.	38
	23d	Discuss implications of the results for practice, policy, and future research.	37
**OTHER INFORMATION**			

Section and Topic	Item #	Checklist item	Location where item is reported
Registration and protocol	24a	Provide registration information for the review, including register name and registration number, or state that the review was not registered.	49-52
	24b	Indicate where the review protocol can be accessed, or state that a protocol was not prepared.	Appendix
	24c	Describe and explain any amendments to information provided at registration or in the protocol.	N/A
Support	25	Describe sources of financial or non-financial support for the review, and the role of the funders or sponsors in the review.	40
Competing interests	26	Declare any competing interests of review authors.	40
Availability of data, code and other materials	27	Report which of the following are publicly available and where they can be found: template data collection forms; data extracted from included studies; data used for all analyses; analytic code; any other materials used in the review.	40
